# Synthesizing Global and Local Datasets to Estimate Jurisdictional Forest Carbon Fluxes in Berau, Indonesia

**DOI:** 10.1371/journal.pone.0146357

**Published:** 2016-01-11

**Authors:** Bronson W. Griscom, Peter W. Ellis, Alessandro Baccini, Delon Marthinus, Jeffrey S. Evans, Ruslandi

**Affiliations:** 1 The Nature Conservancy, 4245 N Fairfax Dr., Arlington, Virginia, United States of America; 2 Department of Biology, James Madison University, Harrisonburg, Virginia, United States of America; 3 The Woods Hole Research Center, 149 Woods Hole Road, Falmouth, Massachusetts, United States of America; 4 The Nature Conservancy, Graha Iskandarsyah 3rd Floor Jl. Iskandarsyah Raya No. 66 C Jakarta, Indonesia; 5 The Nature Conservancy, Fort Collins, Colorado, United States of America; 6 Department of Zoology and Physiology, University of Wyoming, Laramie, Wyoming, United States of America; 7 University of Florida, Gainesville, Florida, United States of America; DOE Pacific Northwest National Laboratory, UNITED STATES

## Abstract

**Background:**

Forest conservation efforts are increasingly being implemented at the scale of sub-national jurisdictions in order to mitigate global climate change and provide other ecosystem services. We see an urgent need for robust estimates of historic forest carbon emissions at this scale, as the basis for credible measures of climate and other benefits achieved. Despite the arrival of a new generation of global datasets on forest area change and biomass, confusion remains about how to produce credible jurisdictional estimates of forest emissions. We demonstrate a method for estimating the relevant historic forest carbon fluxes within the Regency of Berau in eastern Borneo, Indonesia. Our method integrates best available global and local datasets, and includes a comprehensive analysis of uncertainty at the regency scale.

**Principal Findings and Significance:**

We find that Berau generated 8.91 ± 1.99 million tonnes of net CO_2_ emissions per year during 2000–2010. Berau is an early frontier landscape where gross emissions are 12 times higher than gross sequestration. Yet most (85%) of Berau’s original forests are still standing. The majority of net emissions were due to conversion of native forests to unspecified agriculture (43% of total), oil palm (28%), and fiber plantations (9%). Most of the remainder was due to legal commercial selective logging (17%). Our overall uncertainty estimate offers an independent basis for assessing three other estimates for Berau. Two other estimates were above the upper end of our uncertainty range. We emphasize the importance of including an uncertainty range for all parameters of the emissions equation to generate a comprehensive uncertainty estimate–which has not been done before. We believe comprehensive estimates of carbon flux uncertainty are increasingly important as national and international institutions are challenged with comparing alternative estimates and identifying a credible range of historic emissions values.

## Introduction

**Fig 1:**
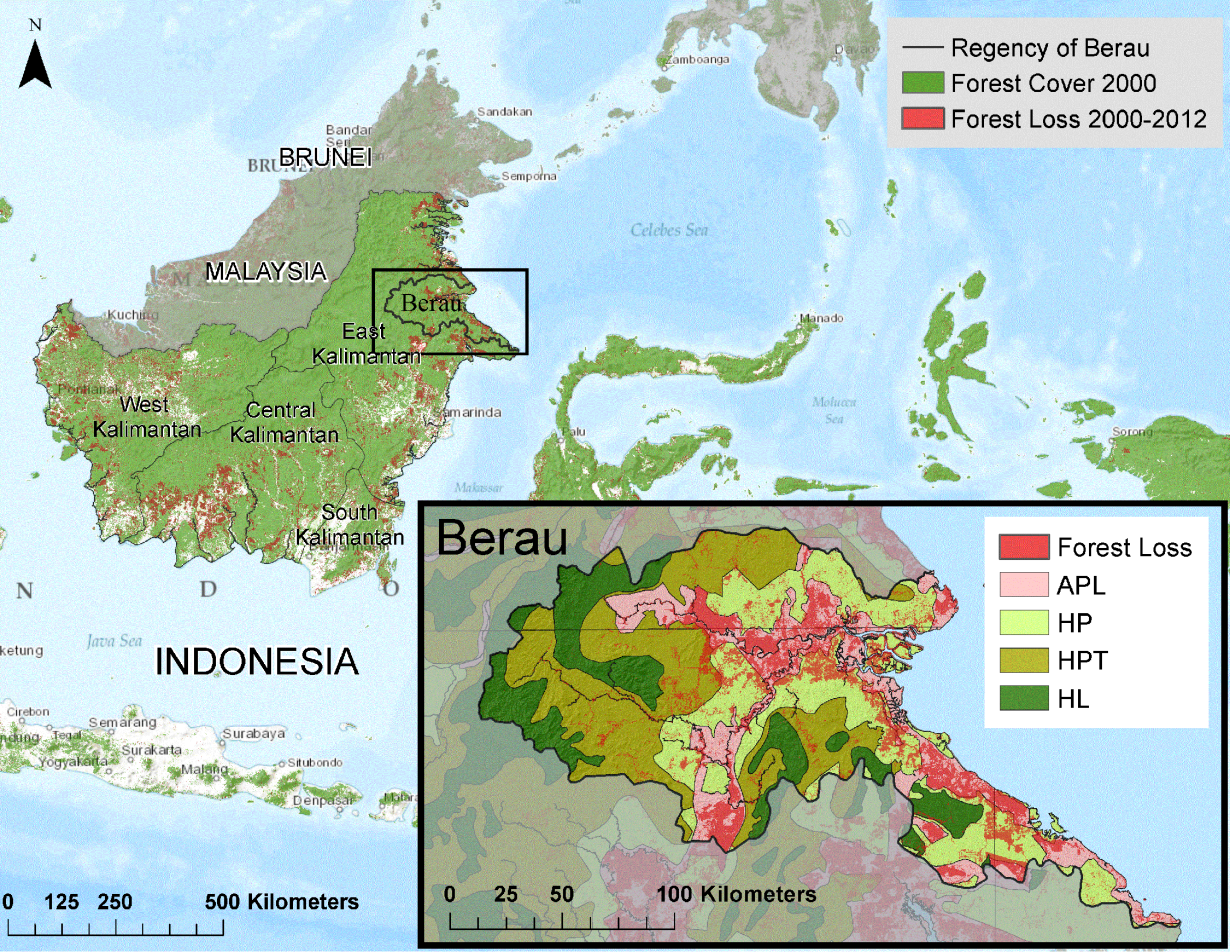
Forest loss in Berau from 2000–2012 as detected by Hansen et al. (2013) is depicted in red (25% canopy cover threshold). Remaining forests are shown in green. Forest loss in Berau is associated with multiple land uses. Oil palm and other agriculture mostly occurs in zones designated for “non-forest” (APL). Fiber tree plantations occur in HP zones. Commercial selective logging concessions are located in HP and HPT zones. Small scale swidden-fallow agriculture is dispersed throughout the landscape. Large coal mining permits overlap all zones except protection forests (HL). Source for spatial plan: Global Forest Watch accessed on February 20, 2014. www.globalforestwatch.org.

Tropical forest conservation could mitigate up to 1/3 of anthropogenic greenhouse gas emissions, and is necessary–alongside ambitious fossil fuel emissions reductions–to stabilize global warming within 2°C [1]. Historic forest carbon emissions estimates are the basis for measuring the climate performance of large-scale forest conservation initiatives, such as is underway in the Indonesian Regency of Berau, located in the province of East Kalimantan on the island of Borneo (Fig 1). Such initiatives need reliable estimates of historic emissions in order for forest conservation to offer a credible solution to climate change.

This paper presents an estimate of historic forest emissions for Berau, using methods that are consistent with standard principles of transparency, accuracy, consistency, comparability, conservativeness, and completeness [2,3]. We were particularly concerned with understanding the uncertainty of our emissions estimate in order to assess accuracy, compare our estimate with others, and to allow for conservative adjustments if results are used for setting reference emissions levels. Such adjustments are called for in the context of UNFCCC reporting, and voluntary carbon standards, when uncertainty is above pre-determined thresholds [3]. Understanding the contributions that different variables in the emissions equation make to overall uncertainty allows for prioritizing research needed to better constrain emissions estimates.

Despite the importance of quantifying uncertainty, we are aware of no prior studies on forest carbon emissions that have conducted a comprehensive assessment of uncertainty emerging from all variables in the emission equation. We present a method for doing so, and discuss the implications for accuracy, comparability with other estimate for Berau, and research needs.

We were interested in assessing the relative accuracy of global datasets on activity and carbon stocks at the scale of Berau because global datasets can offer advantages over regional datasets in transparency, comparability, and consistency. Published global datasets, to the extent they perform well at jurisdictional scales, also have the advantage of more affordable scaling [4–6]. We were interested in demonstrating a historic forest carbon accounting approach in a sub-national jurisdiction because this scale of analysis is critical for implementing and measuring reduced emissions from deforestation and forest degradation, and sequestration from forest growth (REDD+). National and global scales of analysis for measuring REDD+ have received considerable attention in the scientific literature [5,7–10]. In contrast, we are aware of no recent peer-reviewed publications reporting forest carbon accounting targeting sub-national jurisdiction scales in the tropics, yet sub-national initiatives to implement REDD+ are increasingly important [11].

Sub-national jurisdictions have emerged as the proving ground for linking national and global REDD+ processes with the stakeholders and activities that achieve REDD+ on the ground [12]. Sub-national jurisdictions are small enough to link carbon accounting with the strategies and stakeholders that will make lasting changes, but sub-national jurisdictions are also large enough to capture complex landscape dynamics like the displacement of activities in space (leakage) and interactions between multiple conservation strategies, tenure regimes, and forest types. If we can achieve affordable and scientifically robust forest carbon accounting at the sub-national jurisdiction scale, there is a clear pathway to national and global accounting that is effectively linked with REDD+ actions.

We selected the jurisdiction of Berau for this study because it offers a priority microcosm of complex tropical forest loss, gain, and degradation dynamics. For the purposes of this study we define “forest” as a minimum area of 30 x 30m with ≥25% tree canopy cover, where trees are ≥5m in height. Forest gain (or regrowth) and loss is thus a crossing of this threshold, as consistent with Hansen et al. [4] and the “natural forest definition” described by Romijn et al. [13]. Likewise, we refer to “degradation” as any loss of forest carbon stocks for forests remaining forests.

Berau’s landscape (Fig 1) is characterized by an active frontier of land use change impacting large tracts of Dipterocarp forests. These forests are among the world’s highest in carbon density [14], sequestration capacity [15], and biological diversity [16]. Berau Regency follows the watershed boundaries of the Segah River and Kelay river watersheds, which flows into the Berau Marine Conservation Area. Forest conservation in the rugged erosion-prone montane headwaters determines the integrity of this reef system–a member of the Coral Triangle diversity epicenter [17].

Berau has been identified by the Government of Indonesia for early action on REDD+ as part of the World Bank Carbon Fund [18], and by The Nature Conservancy (TNC) and others for priority conservation investments in support of the Regency’s Berau Forest Carbon Program (BFCP). We report here an historic forest carbon emissions estimate to demonstrate a model for jurisdictional accounting.

## Methods

### Carbon fluxes and pools

We accounted for both forest loss and regrowth in order to capture the carbon fluxes associated with land use change in Berau’s dynamic frontier landscape. We also included fluxes from forest degradation due to commercial selective logging. Commercial logging concession permit holders (HA) have legal tenure to 45% of Berau’s remaining forests–more than all other permit categories combined (HTI, HGU, PKP2B). We did not include illegal selective logging (i.e. activity outside of HA permits) in our jurisdictional emissions estimate because our field observations indicated that this is a smaller source than legal logging, and we could not afford to implement the methods necessary to measure the extent of illegal logging; however, we do offer a preliminary estimate of the potential magnitude of this source. We ignored other forms of degradation emissions (e.g. fuelwood collection, escaped fires) based on our assumption from field observations that these are relatively small additional sources, and the difficulty of measuring them.

Our emissions estimate includes all pools (harvested wood products, above and below-ground tree biomass, and dead organic matter) with the exception of soil carbon flux for conversion or degradation of forests on oxic soils. Reviews of the literature have concluded no significant net flux of soil carbon due to conversion of forests on oxic soils to land uses without tillage such as plantations and pasture [19,20]. Since the majority of specified conversion in Berau is to plantations, and we do not have information on the extent of tillage associated with other agricultural land uses, we made the conservative assumption of no net oxic soil carbon flux. We did estimate emissions of soil carbon from loss of forests on anoxic soils (i.e. peat and mangroves). When reporting carbon emissions in units of CO_2_ we used the ratio of the molecular weight of CO_2_ to carbon (44/12).

### Accuracy assessments of existing data

We began by conducting a visual comparison of four existing datasets on forest area change in Berau: one publicly available global dataset [4], one dataset commissioned by The Nature Conservancy [21], one dataset commissioned by the German-Indonesian Forests and Climate Change Program, or “Forclime” [22,23], and one publicly available dataset developed by the Indonesian Ministry of Forestry[24]. We then used 10 m resolution imagery [25] to conduct an accuracy assessment on the Hansen et al. [4] and Forclime [22] datasets. We decided not to subject other available datasets to this quantitative accuracy assessment because initial screening revealed extensive missing data due to cloud cover [21,24] and a manual classification methodology that was not designed for consistent detection of forest change [24]. We used the accuracy assessment to look for any evidence that “forest loss” as defined here is different from alternative definitions of “deforestation” as discussed by Romijn et al. [13]. See S1 File for details of our accuracy assessment methods.

To evaluate the accuracy of the latest global forest biomass datasets by Baccini et al. [5] and Saatchi et al. [6] for Berau, we compared them with independent estimates from field inventory plot data we collected in Berau. We tested for spatial correlation between Baccini and Saatchi datasets, and between the pixel values of each dataset and co-located field plot means, using paired Student’s *t*-tests. Field inventory data plot locations included 90 variable radius plots reported by Griscom et al.[26] and 15 plots reported by Pearson et al. [27]. There was a mean of 3.4 plots per co-located 25 ha Baccini pixel, and 6.0 plots per co-located 100 ha Saatchi pixel, due to clustered locations of field plots.

### Development and modification of datasets, and calculation of emissions

Our emissions equation for Berau was elaborated from the IPCC gain-loss emissions equation 2.4 [2]:

ΔC=ΔCl−ΔCg (1)

where

Δ*Cl* = annual carbon stock loss in Mg carbon yr^-1^

Δ*Cg* = annual gain of carbon in Mg carbon yr^-1^

Carbon losses and gains were calculated as follows:

$$\Delta Cl=\left(\sum_{h=1}^{H}\;{AFd}_{h}\;*\;{Cd}_{h}\;*\;{LFd}_{h}\right)+AWd\;*\;SEFd+Asl\;*\;EFsl\;÷tp$$ (2)

ΔCg=Ar*SFr+Asl*SFsl÷tp (3)

where

*AFd*_*h*_ = total forest area lost during reference period 2000–2010, in forest stratum *h* (ha).

*Cd*_*h*_ = above ground live woody carbon stocks in locations of forest loss for forest stratum *h*, from benchmark biomass map (Mg carbon ha^-1^).

*LFd*_*h*_ = forest loss factor, representing above and below-ground forest biomass emissions per ha of forest loss in forest stratum *h*, as a proportion of *Cd*_*h*_. (%).

*AWd* = forested wetland area lost, 2000–2010 (subset of *AF*_*D*_ to estimate anoxic soil C loss) (ha).

*SEFd* = soil carbon emissions per ha of wetland forest loss(Mg carbon ha^-1^).

*Asl* = forest area legally logged during reference period 2000–2010 (ha).

*EFsl* = above and below-ground forest biomass emissions per unit ha legally logged (Mg carbon ha^-1^).

*Ar* = area of forest regrowth over the reference period (2000–2010) (ha).

*SFr* = above and below ground live woody carbon sequestration rate per unit area of forest regrowth (Mg carbon ha^-1^).

*SFsl* = above and below ground live woody carbon sequestration rate per unit area logged (Mg carbon ha^-1^).

*tp* = reference period = 10 (years).

*h =* forest stratum, or forest biomass classes (N = 13), as described in Table A in S3 File.

**Fig 2:**
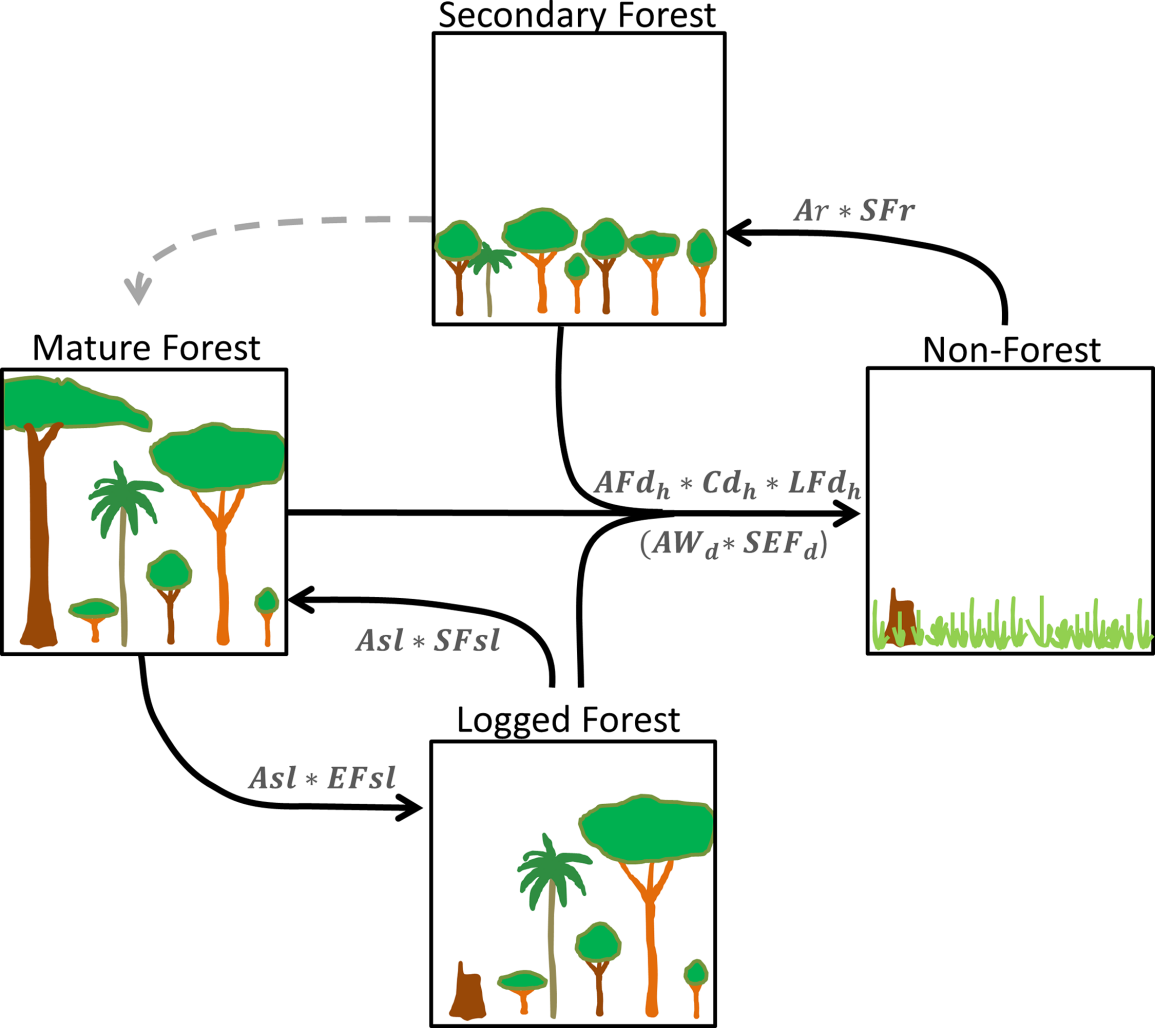
Our forest carbon emissions equation includes (i) activity data variables: forest area lost (*AFd*_*h*_), area of forest legally logged (*Asl*), area of forest regrowth (*Ar*), and (ii) emissions/sequestration factor variables: emissions per unit area forest loss (*Cd*_*h*_ * *LFd*_*h*_), emissions per unit area legally logged (*EFsl*), sequestration per unit area of forest regrowth (*SFr*), and sequestration per unit area logged (*SFsl*). Soil emissions were only included for loss of forested wetlands (***AW***_***d***_ * ***SEF***_***d***_). We assumed that transitions from secondary forest to mature forest (dashed arrow) played a minimal role in carbon flux accounting for this early frontier landscape.

These parameters are depicted in Fig 2 as expressing the major forest loss, gain, and degradation transitions occurring in Berau. We describe methods used to estimate each of these eight parameters in the following sections. See S2 File for a more detailed version of our carbon flux equation.

#### Forest gain-loss activity data (AFd_h_, AWd, Ar)

Historic forest gain and loss rates were calculated using the publically available Hansen et al. dataset [4] (hereafter also referred to as the “Hansen dataset”) over the period 2000–2010. Because gain data is not annual, we assumed the same annual rate of gain for the reference period (2000–2010) as for the source data period (2000–2012).

We stratified all Hansen loss pixels into Oil Palm, Mining, Fiber Plantation, and Agriculture/other disturbance categories through on-screen inspection of available SPOT imagery (10-m resolution, 2009) and Landsat 8 Imagery (2015). If Oil Palm or Mining disturbance was detected outside the bounds of an HGU or PHP2B permit (in space or time), this was noted and tracked accordingly. Within HTI plantation permits, we assumed that forest loss pixels primarily represented conversion to *Acacia mangium* (Willd.) fiber plantations—and hence were classified as “fiber plantations.” Our field observations confirmed this assumption. Resulting disturbance strata were used to assign pixels to different post-clearing emissions scenarios, as described below.

#### Benchmark biomass map (*Cd*_*h*_)

**Fig 3:**
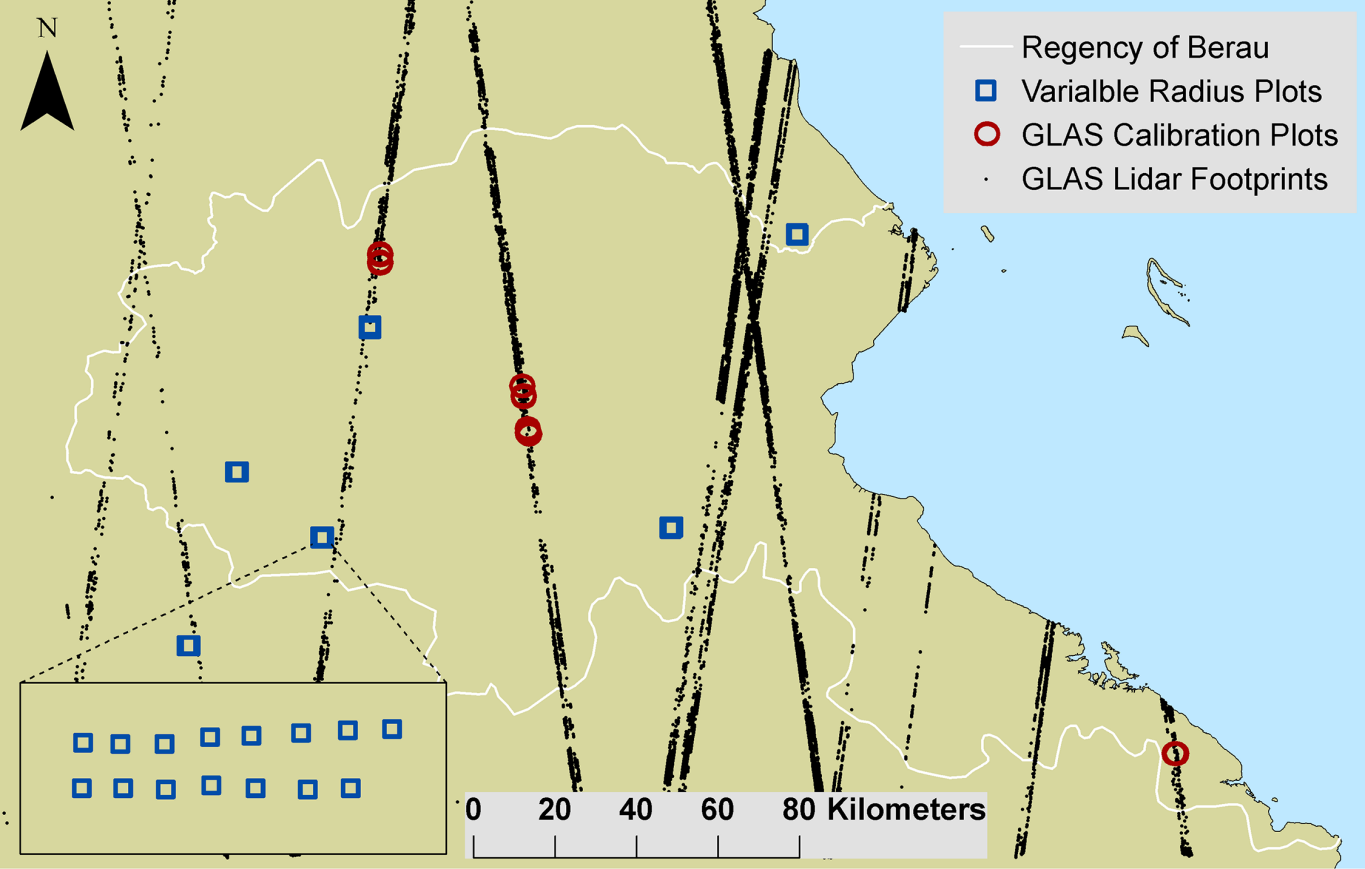
Location of aboveground forest biomass samples. Black dots indicate the location of GLAS lidar footprints (N = 7,574). Red circles are locations of co-located 40 x 40 m calibration plots (N = 15) in Berau (60 more calibration plots were located in SE Asia). Blue squares indicate the location of transects of variable radius plots (N = 80).

We developed a benchmark biomass map for Berau using a “stratify and multiply” approach [28]. We generated a regionally-calibrated version of the pantropical GLAS biomass model developed by Baccini et al.[5], based on 60 40x40 m field plots they collected in Indonesia (N = 40) and in Vietnam (N = 20), and an additional 15 plots collected in Berau as part of this study. Plots were co-located with GLAS footprints (Fig 3). The field plot inventory design (all trees ≥ 10 cm dbh) is as described by Baccini et al.[5]. This generated aboveground forest biomass estimates for 7,574 GLAS footprint estimates within Berau with >25% canopy cover in 2000 and no intervening forest loss between 2000 and 2009 (the year after last GLAS footprints were collected) as mapped by Hansen et al.[4].

This large number of biomass estimates allowed us to conduct an analysis to identify optimal forest biomass strata for Berau. To do so, we acquired four spatial datasets to explain the distribution of forest biomass within Berau: a dataset differentiating “primary” from “non-primary” forests [29], a soil-based “RePPProT land systems” dataset [30], a map of peatlands [31], and a 30x30 m elevation dataset[32].

We simplified the RePPProT dataset into seven major substrate groups: acidic, alluvial peat, alluvial dense peat, coastal peat, mangrove, karst, and metamorphic/volcanic. We were limited to using this substrate terminology reported by RePPProT, rather than soil taxonomy terminology, due to the absence of a soil map at the relevant scale. In order to translate the continuous elevation raster dataset into a limited set of strata, we defined elevation classes as a function of forest type/structure at thresholds described at 300 m [33], 900 m (used to divide montane and lowland WWF ecoregions [34]), and 1500m [35]. We added a threshold at 100m (approximate distinction between “plains” and “hills” in the RePPProT dataset) to achieve an area-balanced elevation stratification, since 75% of Berau’s forest area lies below 300m. The secondary forest stratum was mapped to pixels detected as forest loss by Hansen et al.[4], yet classified as non-forest in 2000 [29].

We used GLAS footprint estimates to confirm or deny a statistical basis (using one-way ANOVA with Tukey’s multiple comparison test) to maintain the strata described above as separate or lumped biomass classes. We also used the GLAS footprint biomass estimates to compare (ANOVA) the set of forest biomass classes in our benchmark biomass map with two alternative systems for stratifying forest biomass in Berau: (i) Indonesian Ministry of Forestry Land Cover map [24] and (ii) a new forest biomass stratification by Navratil [22]. We used an independent set of forest inventory variable radius plots (N = 80) in Berau, described by Griscom et al.[26], to validate GLAS-based biomass strata means (Fig 3). See S3 File for methods details.

#### Forest loss factor (LFd_h_)

This factor was derived from a process-based calculation to model forest loss emissions over the 10-year reference period and included estimates of below-ground biomass fraction [36], above-ground necromass fraction [37], combustion completeness in burned areas [38], charcoal elemental fraction[39], wood product life cycle [40], round wood biomass fraction extracted from land clearing [26], wood density [41], and carbon fraction [42]. We calculated a post-deforestation wood decay factor (percent of dead wood decayed per year) for all forest strata using a linear decay rate of 0.24 [43], and modelling decay emissions based on the amount of dead wood created by deforestation year by year for the reference period. To calculate post-logging decay, the same linear rate was modelled based on wood harvest rates during the reference period. See S2 File for the complete list of parameters used in our emissions equation.

#### Soil carbon emissions factor (SEFd)

Our estimates of soil carbon emissions from loss of wetland forests (peat forests and mangroves) assume a predominance of “heavier activities” such as conversion to aquaculture as indicated by MacKinnon et al. [16]. In the case of mangrove loss, we followed Donato et al.’s [44] “high end” estimate which assumes 75% organic soil carbon loss from top 30 cm of soil profile, and 35% loss from remainder. In the case of peat forests, our review of FIRMS data [45] and our field observations suggest that nearly all cleared peat forests are subsequently burned. Therefore, we assumed 100% soil carbon loss of drained peat in forest loss areas. Peat drainage depth was estimated at 60cm following Hooijer et al.*’s* [46] average for “typical small scale agricultural areas”, a conservative assumption given the likely clearing of at least 1 deeper-draining peat-based industrial plantation in Berau during the reference period. Peat soil depths and carbon densities were estimated from Wayunto et al. [31].

#### Logging emissions factor (EFsl)

Our logging emissions factor (*EFsl*) is derived from field inventory data collected in and near Berauto estimate the volume of live above- and below-ground tree biomass lost to necromass due to felling and skidding [26]. Because haul road construction results in a complete loss of forest cover, hauling emissions was estimated using activity data from the Hansen dataset(described below) and the same calculation used for forest loss ($\sum_{h=1}^{H}\;{AFd}_{h}*{Cd}_{h}*{LFd}_{h}$ from Eq 2). We generated a preliminary estimate of illegal logging emissions based off of our logging emissions factor, as described in S4 File.

#### Logging activity (Asl)

We concluded that it was not feasible to build a remote-sensing dataset of selective logging felling and skidding activity. We did use remote sensing (Hansen dataset), to detect area of hauling activity (logging haul roads and log landings). We confirmed that this dataset conservatively estimated the extent of newly built haul roads mapped with GPS by Griscom et al. [26]. We segregated the linear dendritic pattern of this logging haul road activity from forest loss associated with conversion activities (e.g. agriculture, plantations) with a heads-up digitization process that reclassified all Hansen dataset loss pixels in licensed logging concessions 3 pixels wide or less (90 m) as “logging roads.”

We acquired all available government records of logging activity (area and volume data for 57% of all HA permit years) between 2000 and 2012 across the 20 permitted selective logging concessions in Berau, and used this to calculate an average annual legal harvest rate for the reference period. See S4 File for details.

#### Sequestration factors (SFr, SFsl)

We assigned a rate of additional forest carbon sequestration in response to commercial logging (*SFsl*) of 0.368 MgC ha^-1^ based on measurements of tree growth rates prior to and 8 years after commercial logging in experimental forestry research plots established in 1990, covering 48 ha in Berau [47]. We assigned three different values for the rate of secondary forest growth (*SFr*) occurring in Hansen regrowth pixels, as a function of three types of secondary forest we have observed in Berau. In areas with recorded fiber plantation permits (HTI, Fig 1) we assigned a sequestration rate of 9.8 MgC ha^-1^ yr^-1^, the mean of reported growth rates reported for *Acacia mangium* (Willd.) in Borneo [48,49]—the fiber plantation species observed in Berau. In oil palm permit areas (HGU), we assigned a sequestration rate of 2.97 MgC ha^-1^ yr^-1^ for oil palm (*Elaeis guineensis* Jacq.) based on a study by Thenkabail et al. [50]. For regrowth pixels occurring in all areas outside these permit types we assigned a sequestration rate of 3.85 MgC ha^-1^ yr^-1^, based on a pantropical review of native forest growth rates [51] for wet tropical forests (>1700 mm rainfall, without severe dry season).

#### Comprehensive analysis of uncertainty

To estimate uncertainty of our results, we conducted Monte Carlo simulation of our emissions equation using defined distributions for all of the input parameters in our carbon flux equation (S2 File), building on methods described by Morton et al.[52]. The Monte Carlo method is endorsed by the Intergovernmental Panel on Climate Change (IPCC) as a reliable and more advanced (Tier 2–3) approach to accounting for uncertainty [53], and is often used for uncertainty propagation (e.g. [54]). In order to derive distributions, we separated our input parameters into two categories: 1) those with sufficient data to define standard errors at the geographic scale of this study, and 2) those without sufficient data to calculate standard errors. For all input parameters in the second category, we assigned a default uncertainty value (percent error at ±95% CI from the mean) based on our assessment of the range of uncertainty (Table 1). In doing so, we were conservative in assigning percent error values at the upper end of the assessed range. We considered all available evidence, including our review of the global literature and the geographic context of the study, when assessing the range of uncertainty. See S2 File for source data and literature reviewed as well as the uncertainty category and uncertainty value for each parameter. See S5 File for R-code used to run the Monte Carlo simulation.

**Table 1:** Uncertainty categories for emissions equation parameters.

Uncertainty category	Assessed range of percent error	Assigned percent error
Very High	> 50%	100%
High	20–50%	50%
Medium	10–20%	20%
Low	<10%	10%
Unassigned	derived from sample data	

Note: Errors are expressed as percent error at ±95% CI from the mean. Unassigned uncertainty values were derived from distributions defined with empirical data.

Applying the central limits theorem, we converted 95% confidence intervals to standard errors of the mean (SEM) by dividing by 1.96. Input parameter SEMs were used to define distributions used in Monte Carlo simulations ([55]1,000,000 iterations). We randomly drew a single value from defined distributions, for each parameter and at each simulation, and fed values into the emissions equation to calculate 95% confidence intervals for our total net forest carbon emissions estimate, and other flux estimates (gross deforestation, gross degradation, wetland soil, sequestration).

To evaluate the source contribution of different input parameters to overall net emissions uncertainty, we grouped parameters based on their carbon pool and/or flux mechanism and re-ran the Monte Carlo simulation, replacing input parameter distributions for each group with mean values and observing the percent reduction in net emissions uncertainty. The resulting uncertainty reductions indicate the relative contribution from each group of parameters to overall uncertainty.

#### Comparison with alternative emissions estimates for Berau

We compared our estimate with three alternative estimates for Berau. Two estimates were publicly available [18,22]. We generated two additional estimates for Berau from publicly available tools [56,57]. We followed default settings and datasets associated with each of these tools. In selecting options presented, we made choices and selected inputs to be consistent with our methods for this study.

## Results

### Emissions

**Fig 4:**
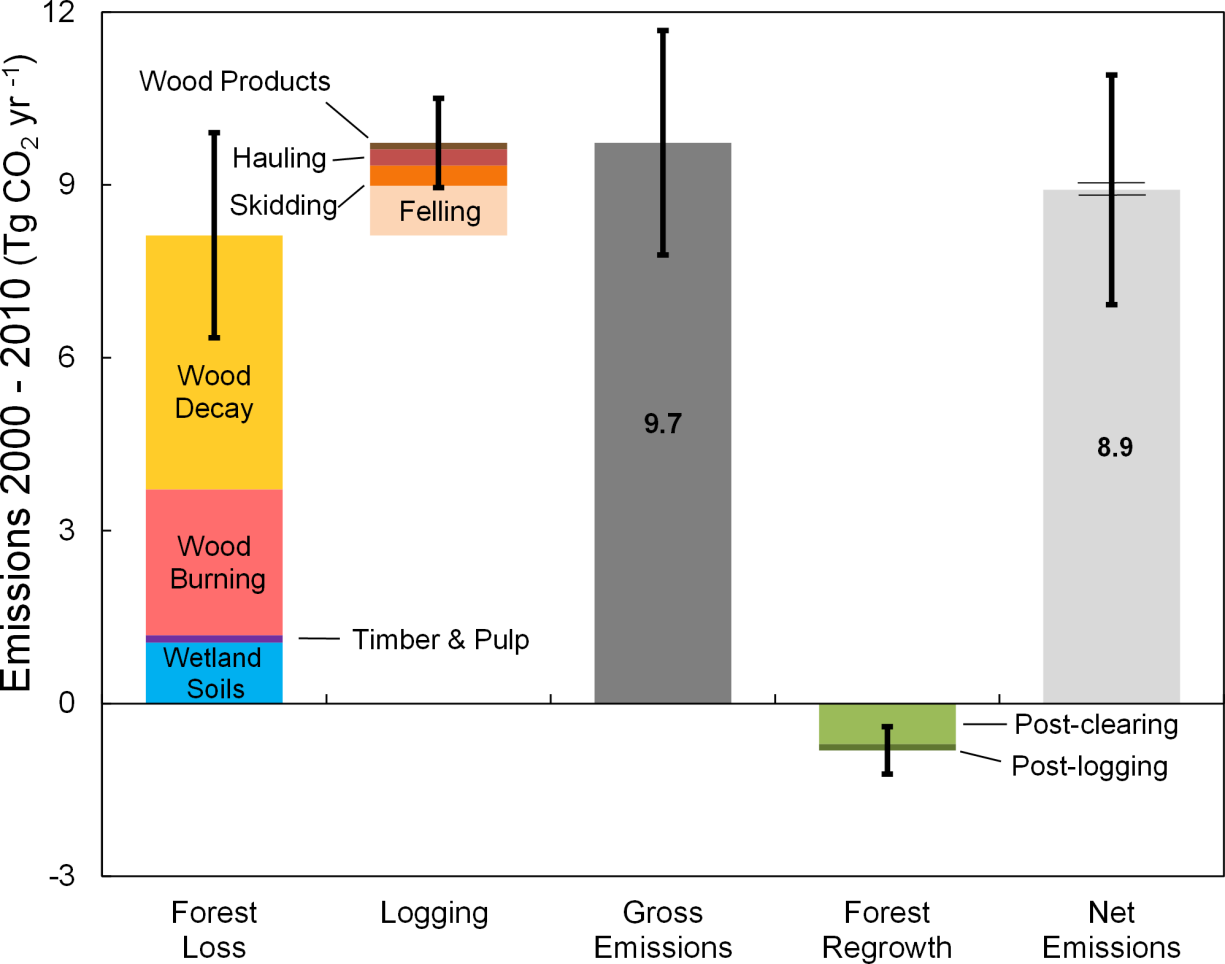
Gross and net emissions, in millions of tonnes (Tg) of CO_2_, are represented according to forest loss, logging, and sequestration components. Values represent annual means during the 2000–2010 period acrossBerau. The wider bars just above and below the top of the net emissions column represent the width of error bars (±1%) if we had only accounted for uncertainty in our activity dataset and map-based uncertainty in our biomass dataset.

Berau generated 8.91 ± 1.99 million metric tons (Tg) of net annual CO_2_ emissions per year during 2000–2010 as a result of forest loss and gain, and legal logging activities (Fig 4). This net value reflects gross CO_2_ emissions of 9.73 ± 1.95 Tg yr^-1^ that were 12 times larger than gross sequestration of 0.82 ± 0.41 Tg yr^-1^ over the 2000–2010 period. Gross mean annual emissions from loss of above and below-ground tree biomass in Berau (8.67 TgCO_2_ yr^-1^) represents loss of 0.55% of the total year 2000 above and below-ground forest carbon stocks in Berau (427 TgC). These stocks are dominated by Dipterocarp forests on oxic soils, which cover 95% of Berau’s remaining forest area.

The majority (84%, 8.13± 1.78Tg CO_2_ yr^-1^) of gross emissions was due to forest loss. Of forest loss emissions, the bulk (87%) was from decay and burning of above and below-ground tree biomass and necromass. The remainder was emissions from the decomposition of soil organic material due to the loss of wetland forests (Fig 4). Secondary forests regrowth sequestered 0.71 Tg CO_2_ yr^-1^ and offset 9% of gross forest loss emissions.

Forest degradation due to commercial selective logging in oxic soil Dipterocarp forests generated the other 16.5% of Berau’s gross forest carbon emissions during 2000–2010 (1.60 ± 0.77 Tg CO_2_ yr^-1^). The largest component of logging emissions was caused by felling harvest trees (0.97Tg CO_2_ yr^-1^). These felling emissions were the result of the decay of both harvested tree remainders and non-harvest trees destroyed by felling (collateral damage). The second and third largest components were due to decay of tree biomass resulting from skidding impacts (0.35 Tg CO_2_ yr^-1^) and haul road impacts (0.28 Tg CO_2_ yr^-1^). Decay of wood products removed from the logging concessions represented a small additional source of emissions (0.11 Tg CO_2_ yr^-1^). Additional regrowth in response to logging activity (0.11 Tg CO_2_ yr^-1^) offset 7% of gross logging emissions (Fig 4).

**Fig 5:**
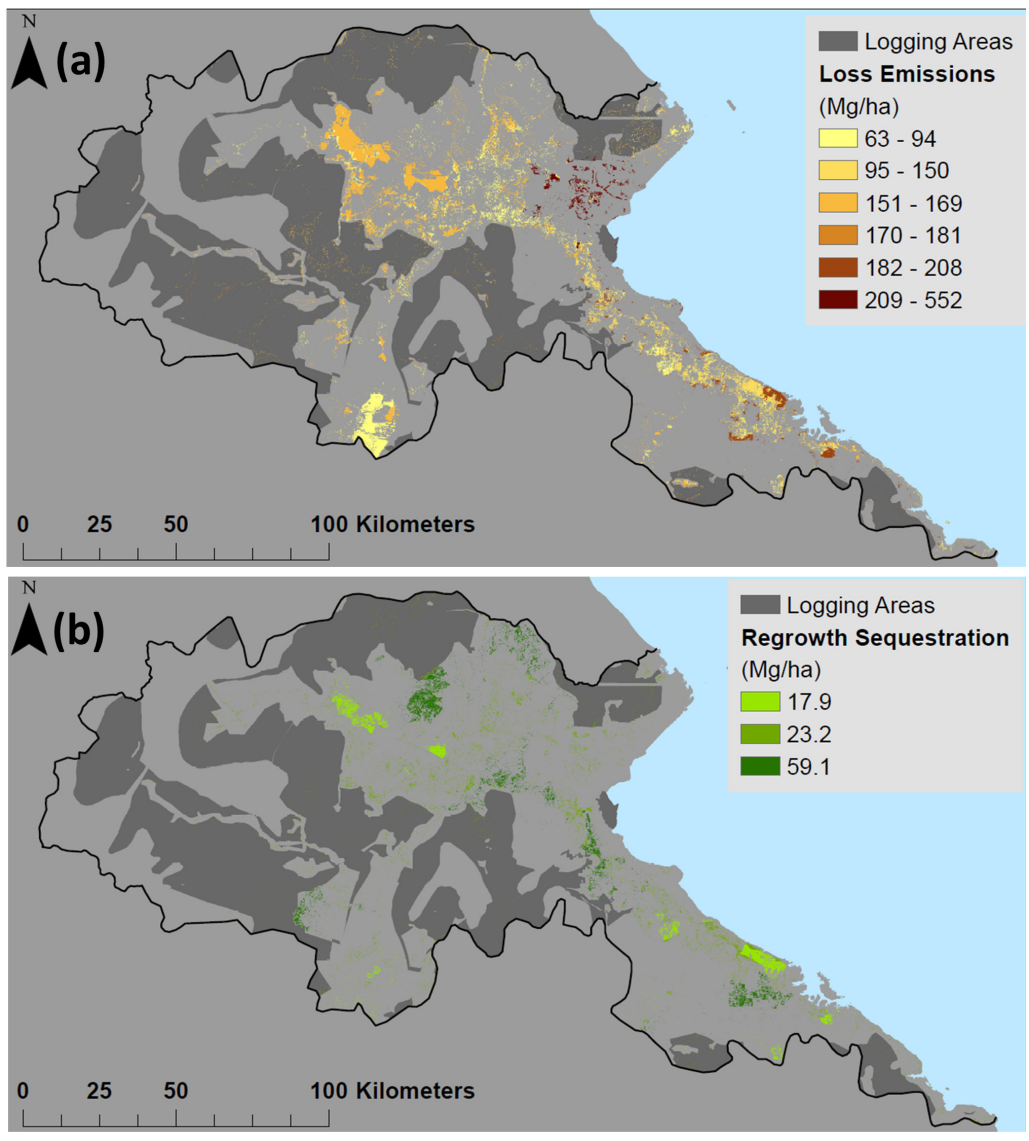
Spatial distribution of forest carbon flux, in MgC ha^-1^, is represented in (a) shades of brown for forest loss emissions, and (b) shades of green for forest regrowth. Monotone grey zones in (a) and (b) represent HA logging concessions–within which logging emissions and regrowth occurred, but specific locations are not known.

Emissions and sequestration reflect a dynamic early frontier landscape in Berau. The frontier of forest loss and regeneration was primarily located in the central and south-eastern portions of the Regency (Fig 5). The highest density of carbon emissions occurred from loss of limited areas of wetland forests near the coast (peat and mangroves). The majority (64%) of net emissions in Berau during 2000–2010 was due to forest loss in APL zones. About half of those emissions occurred in areas with known permits for conversion (Table 2, Fig 1). Forest loss due to oil palm (*Elaeis guineensis*) conversion caused 44% of the emissions from APL zones, nearly all of which were legally permitted. The remainder of conversion was dominated by forms of small scale agricultural, most of which was not associated with known permits. Despite the large area falling within mining permits (PKP2B), only 2% of forest emissions were linked to mining activity (Table 2).

**Table 2:** Emissions and area change during 2001–2010 by three spatial plan zones (*in italics*), four permit types, and areas without known permits.

Disturbance Type	Permit Type	Land Area (ha)	Forest Area (ha, 2000)	Area Disturbed (ha yr-1)	Area Forest Regrowth (ha yr-1)	Gross Emissions (Tg CO2 yr-1)	Gross Sequestration (Tg CO2 yr-1)	Net Emissions (Tg CO2 yr-1)	Percent Net Emissions
*"Non-Forest" (APL)*		*511*,*904*	*382*,*773*						
Oil Palm	HGU	206,683	194,513	4,509	1,379	2.38	0.130	2.25	25.3%
Oil Palm	None			477	74	0.27	0.007	0.26	2.9%
Agriculture / Other	None			3,786	1,306	2.79	0.313	2.48	27.8%
Agriculture / Other	HGU	206,683	194,513	1,050	328	0.56	0.031	0.53	5.9%
Mining	PKP2B	117,745	102,312	351	79	0.18	0.007	0.17	2.0%
Mining	None			111	15	0.05	0.001	0.05	0.6%
*Production Forest (HP/HPT)*		*1*,*322*,*887*	*1*,*248*,*355*						
Skidding/Felling	HA	898,725	888,438	8,654	8,654	1.32	0.076	1.25	14.0%
Logging Roads	HA	898,725	888,438	690	259	0.28	0.024	0.25	2.8%
Fiber Plantation	HTI	266,351	242,322	2,025	2,029	1.02	0.191	0.82	9.2%
Agriculture / Other	HA	898,725	888,438	1,380	314	0.79	0.030	0.77	8.6%
Mining	HTI	266,351	242,322	104	20	0.06	0.002	0.06	0.7%
*Protection Forest (HL)*		*359*,*979*	*358*,*866*						
Agriculture / Other	None			65	49	0.04	0.00	0.03	0.4%
**Total**		**2,194,770**	**2,102,255**	**23,203**	**14,506**	**9.73**	**0.82**	**8.91**	**100%**

Notes: Within APL (= non forest area) zones, HGU is a permit granted for specific land use activities. PKP2B is a coal permit that can overlap with other permit types. Production forest zones, designated mainly for sustainable forest production, include limited production forest (HPT) and permanent production forest (HP). IUPHHK-HA (= HA) permits for selective logging harvest are granted within HPT and HP, while IUPHHK-HTI (= HTI) permits for conversion to plantations are granted within HP. We assumed all forest loss in HTI permits represented conversion to fiber plantations, but some may have represented agricultural conversion. Totals may not match sum of individual row values, due to alignment problems with permit and zone boundaries. Our analysis does not reflect the 2014 allocation of lands within HP and HPT to a new category of convertible production forest (HPK).

Virtually all of the remaining net forest emissions in Berau (35%, 3.15 Tg CO_2_ yr^-1^) were from production forest zones (HP, HPT). About half of those emissions were from logging activity. The other half were from conversion of native forests to fiber plantations (*Acacia mangium*), small scale agricultural, and mines (Table 2). Only 0.4% of net forest emissions were due to forest loss in protection forests (HL).

**Fig 6:**
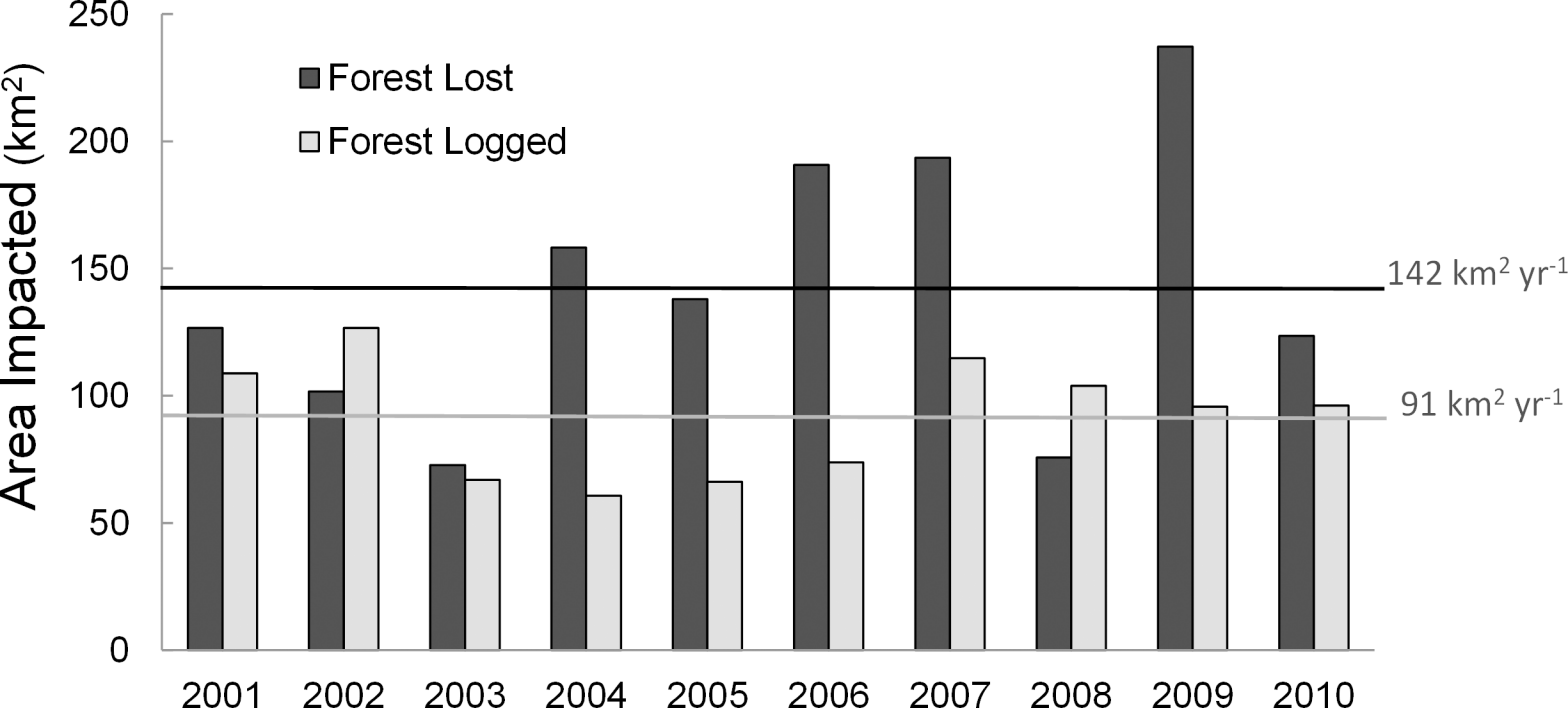
Extent of forest loss and legal selective logging through time (2001–2010) in Berau. Black and grey lines represent mean annual forest loss and forest logged, respectively. There was not a significant linear trend for either forest loss or for logging activity during this ten year period (P>0.05).

We did not detect a significant linear trend during the ten year historic reference period of 2001–2010 for forest loss (R^2^ = 0.141, F (1, 8) = 1.310, P = 0.285) or logging activity (R^2^ = 0.000, F (1, 8) = 0.003, P = 0.957, Fig 6).

### Activity data

We selected the Hansen dataset [4] for forest loss and gain activity data due to its higher accuracy (error of commission = 6.8%, error of omission = 9.2%) as compared with the best available alternative (Forclime dataset [22]: error of commission = 12.8%, error of omission = 22.7%) and other advantages as compared with alternative datasets (see S1 File for details). Our accuracy assessment also confirmed our assumption that the Hansen dataset represents anthropogenic forest loss activity in Berau (we found no evidence of natural disturbance events detected as forest loss).

On average, 9,344 ha yr^-1^ (297,766 m^3^ yr^-1^) were legally harvested for timber in Berau within HA concession permits between 2000 and 2012, based on government records (see S4 File for details). We also report in S4 File a preliminary estimate of additional illegal logging activity, although we did not include it in our emissions estimate due to methods limitations.

### Biomass dataset

We did not detect a spatial correlation between forest biomass using field plots in Berau (N = 105) provided by Griscom et al.[26] and Pearson et al.[27] and pixel values of what we considered the best available pantropical forest biomass datasets: (i) Baccini et al. [5] (R^2^ = 0.0006, F (1, 32) = 0.017, P = 0.90), and (ii) Saatchi et al. [6] (R^2^ = 0.0007, F (1, 18) = 0.011, P = 0.92). The degrees of freedom in these statistics varied as a function of the number of field plots that fell within (and thus were averaged within) a given pixel (mean of 6.0 field plots per Saatchi pixel, and 3.4 field plots per Baccini pixel).

Since we were not able to confirm or quantify the accuracy of these pantropical biomass datasets at the scale of Berau, we decided to build a new “stratify and multiply” forest biomass dataset for Berau (as described in the methods section). The relationship between co-located field plots and our GLAS modeled footprint estimates ((R^2^ = 0.37, F (1, 73) = 92.30, P = >0.001) is depicted in Fig A in S3 File.

**Fig 7:**
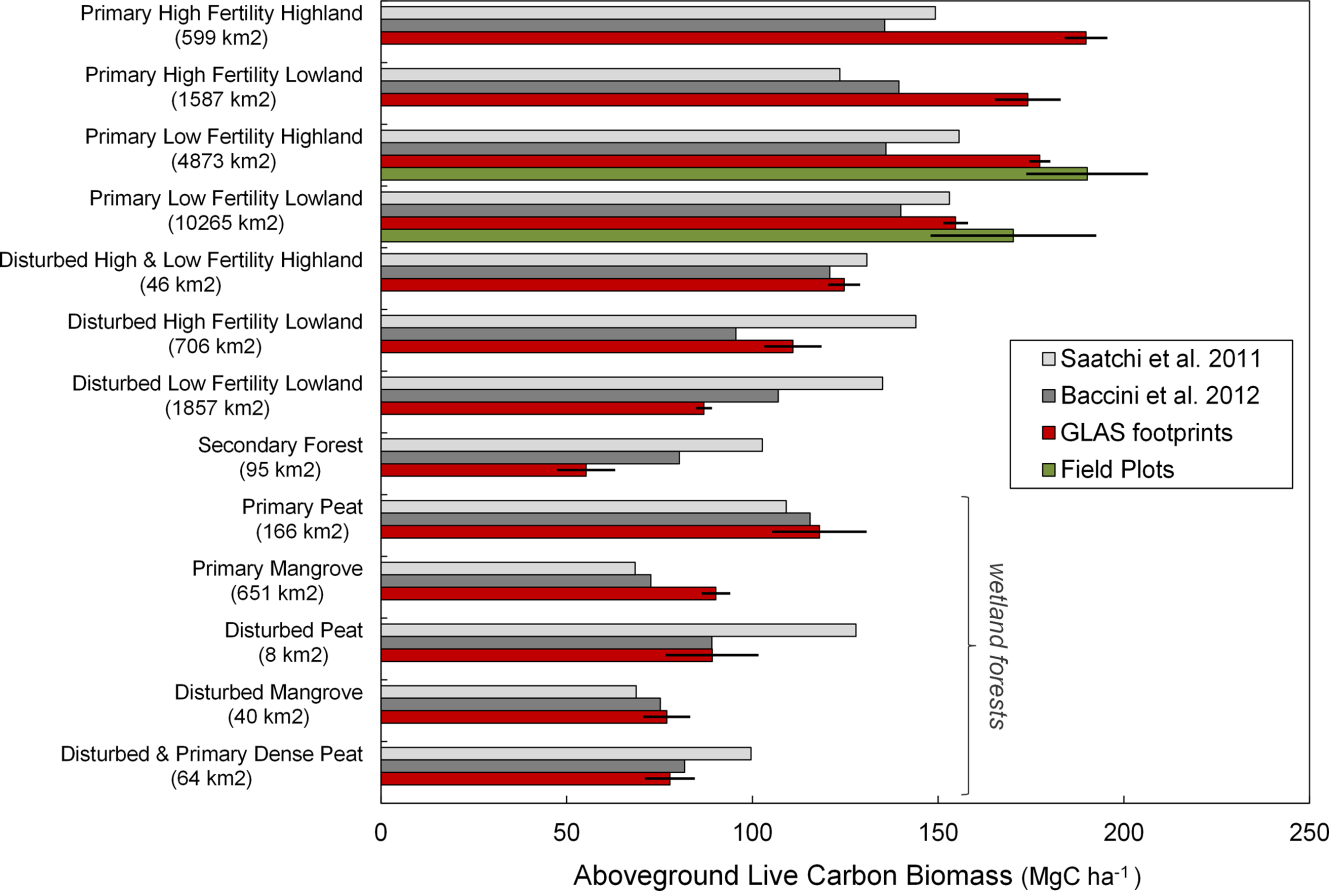
Aboveground carbon stocks in 13 forest biomass classes. Red bars depict mean aboveground biomass (MgC ha-^1^) for the disturbance-elevation-soil forest biomass classes we derived using GLAS footprint estimates (N = 7573). For most classes, mean biomass derived from the latest pantropical forest biomass datasets [5,6] did not fall with 95% confidence intervals (error bars) from the GLAS-derived means. GLAS-derived means for the two most extensive biomass classes (primary low fertility highland and lowland) did overlap with 95% confidence intervals independent variable radius field plot means (green bars). See S3 File for information on carbon stocks, area change, and percent emissions by class.

**Fig 8:**
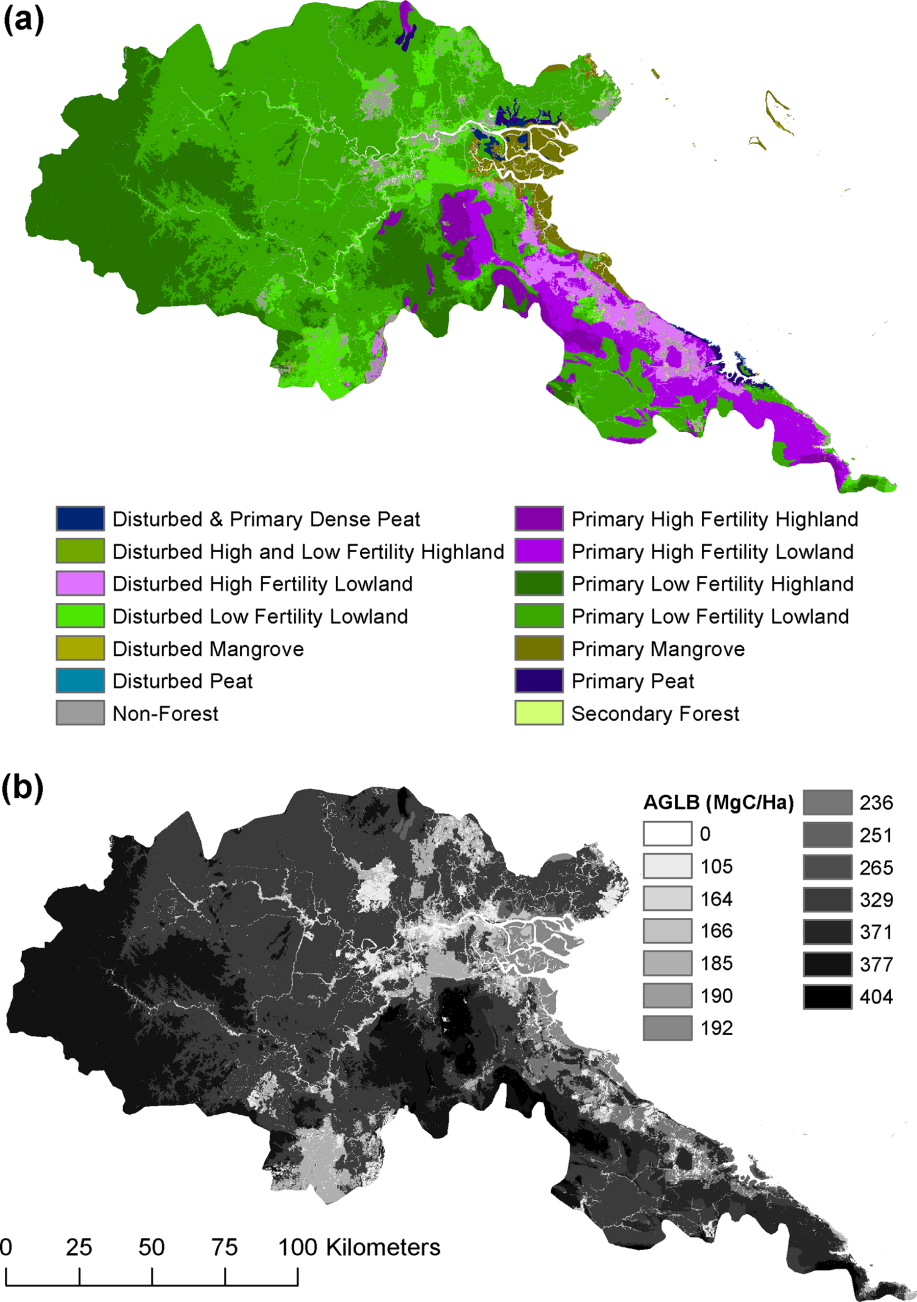
Spatial distribution of forest biomass and biomass classes. We developed a new forest biomass map for Berau (a) using mean values of GLAS footprint estimates assigned to each disturbance-elevation-soil forest biomass class (b). See Table A in S3 File for details on the derivation of biomass classes.

We used GLAS footprint estimates as the basis for calculating average values for our biomass classes (Fig 7), as they are distributed across Berau (Fig 8). The derivation of biomass classes, and related findings about the factors explaining forest biomass in Berau, are described in S3 File. Our GLAS-based biomass estimates for the two most extensive forest biomass classes were corroborated by the sample of dual-prism field plots reported by Griscom et al. [26], as shown in Fig 7.

Based on our comparison of alternative forest biomass classification systems, we proceeded to use our disturbance-elevation-soils biomass classes in calculating emissions, after confirming that analysis of variance (Minitab® Statistical Softwarev16.2.2) among classes (F (1,12) = 311.03, P < 0.001) was stronger than that of the equivalent number of Indonesian Ministry of Forestry classes (F (1,12) = 253.00, P < 0.001) or Navratil (2013) classes (F (1,12) = 116.40, P < 0.001) with respect to our GLAS biomass estimates. Above-ground live biomass was highest for primary forests on oxic soils, intermediate for disturbed forests, and lowest for secondary forests. Highland oxic soil forests (>100 m) supported higher biomass than lowland oxic soil forests. More basic “high fertility” oxic soils (karst, metamorphic, and volcanic) supported higher biomass forests than more acidic “low fertility” soils. Wetland forests (anoxic soils) had lower aboveground biomass compared with oxic soil forests, for a given disturbance class (Fig 7, and see S3 File for details).

### Analysis of Uncertainty

Overall uncertainty (95% confidence intervals) of our net emissions estimate was ±29% of our net emissions estimate reported above. Forest loss activity and aboveground live biomass contributed < 1% and 4% respectively of overall uncertainty in our emissions estimate. The largest sources of uncertainty were from decay rate (30%), wetland soil carbon (17%), and area of regrowth (11%) (Table 3).

**Table 3:** Contributions to overall uncertainty in forest carbon flux estimate for Berau.

Sources of uncertainty	Percent contribution to overall uncertainty
Decay	30%
Wetland soil carbon	17%
Regrowth area	11%
Regrowth rate	8%
Logging	8%
Fire	6%
Other	7%
Aboveground live C	4%
Necromass	4%
Roots	4%
Forest loss	0.27%
Total	100%

Note: For parameters involved with “other” sources of uncertainty see S2 File.

### Comparison with other emissions estimates for Berau

**Fig 9:**
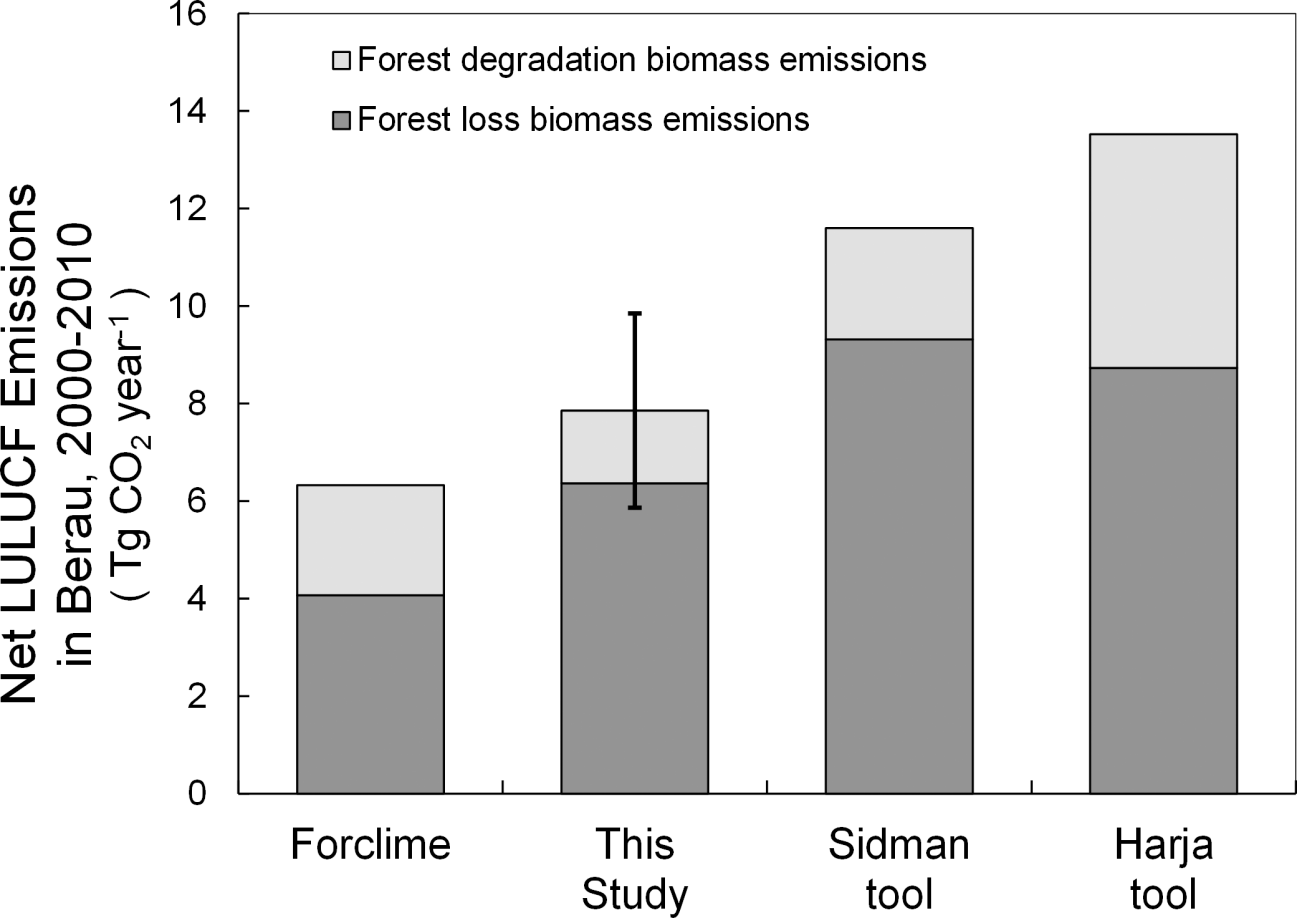
Comparison of alternative emissions estimates for Berau. Two alternative historic emissions estimates for Berau were higher than the upper end of our modeled uncertainty range (error bars are 95% confidence intervals). Those two estimates are from our use of emissions tools by Sidmanet al.[56] and Harjaet al.[57]. A fourth estimate by Forclime[22] fell below ours and within our uncertainty range.

Alternative historic emissions estimates for Berau derived from emissions calculation tools that are available [54,55] were higher than the upper end of our modeled uncertainty range (95% confidence intervals), while the Forclime [22] estimate fell below ours and within our range of overall uncertainty (Fig 9).

## Discussion

### Alternative emissions estimates for Berau

The implications of the selection of alternative historic emissions estimates for Berau are large: our estimate for the same fluxes during 2000–2010 is 32–42% less than what we derive from two available accounting tools (Fig 9), a difference equivalent to the potential emissions reductions reported by GOI for Berau [18]. This is due to the combination of both higher forest loss and forest degradation emissions estimates generated by these alternative accounting methods. While the difference in degradation emissions could be explained by our decision to ignore illegal logging emissions due to absence of empirical data, it is difficult to explain the large differences in net forest loss emissions given the absence of information about uncertainty associated with other estimates.

Most of the other estimates are above the upper level of our estimated overall range of uncertainty (Fig 9). One emissions estimate [22] falls below ours yet within our confidence intervals–and thus offers a conservative alternative emissions value for Berau (Fig 9). Our accuracy assessment finds that the Hansen dataset slightly underestimates forest loss in Berau. We find that Bellot et al.’s source dataset [22] excessively underestimates forest loss primarily due to missing data associated with cloud cover (error of omission over twice that of Hansen, S1 File). Our accuracy assessment and attribution analysis revealed that Navratil [22] included forest loss from conversion to exotic species plantations as “deforestation.” Thus, a definitional issue of “deforestation” vs “forest loss” does not appear to explain our dataset differences, nor would definitional issues explain why both of our estimates are substantially less than other estimates.

While definitional issues do not appear to explain the differences between our estimate and others, we prefer the term “forest loss” rather than “deforestation” because inconsistent use of the latter term has created confusion. In other geographies, large differences have been found between estimates of “deforestation” as defined by the IPCC (2006) and estimates based on a “natural forest definition” [13]. We suggest that all loss of tree cover over an agreed threshold (e.g. 25%) and unit of measurement (e.g. 30 m pixel) caused by anthropogenic impacts should be included in accounting for “forest loss” (and “deforestation”) emissions. Likewise, any subsequent forest recovery should also be accounted for, rather than assuming that forestry rotations are neutral.

### Proximate Causes of Emissions

Our results also highlight the implications of ignoring emissions from the conversion of native forest to exotic tree plantations based on a restrictive interpretation of the IPCC definition of “deforestation.” Net emissions in Berau were dominated by forest loss (83%) nearly half of which were due to conversion to exotic tree plantations (*Elaeis guianensis* and *Acacia mangium*,Table 2). The remaining emissions due to forest degradation from legal logging (17%) are above the 10% threshold set by the World Bank Forest Carbon Partnership Facility for required tracking of degradation emissions [58].

The proximate causes of forest loss emissions across Indonesia differ from what we found in Berau. Abood et al. [59] report that the largest portion of national forest loss emissions are associated with fiber plantations (35%), followed by oil palm plantations(28%) and logging concessions (22%). In Berau, conversion of forests to oil palm plantations was responsible for the same proportion of overall forest emissions (28%); however, agricultural activities (that we were not able to characterize further) were the largest proximate cause of forest loss in Berau (43%), while fiber plantations (9%) and mining (3%) contributed considerably less (Table 2).

Most of the emissions we report for Berau (59%) were associated with legal permits to convert, or extract timber from, forests. We assume that forest loss emissions occurring outside of known permits (31%) within APL zones were due to illegal activities; however, we were not able to confirm that we acquired all legal permit data (unknown error of omission). Some emissions were due to activities more obviously inconsistent with the spatial plan, and more likely to be illegal. Forest loss within HA permits that did not follow the spatial patterns of logging infrastructure (haul roads and base camps) contributed 9.3% of Berau’s emissions–and appeared to be dominated by illegal small scale agriculture. We found very low emissions in protection forests (HL), which could be due to successful government intervention and/or remoteness and steep terrain.

By year 2010, only 11% of Berau’s land area did not have forest cover, a small proportion relative to the >20% loss of global tropical forest cover since 1960[60].Meanwhile, Berau’s mean annual rate of gross forest loss from 2000–2010 (0.71%) was 60% higher than the pantropical mean (0.45% using [4]). These statistics indicate a dynamic early frontier landscape. Nearly all (96%) of forest loss since 2000 in Berau has occurred on lands zoned for conversion (APL and HP), and nearly half (47%) of the remaining forests in Berau are currently zoned for conversion. Most of the remaining forests zoned for conversion are zoned for fiber plantations (HP, 60%).

### Adapting global data for a jurisdictional scale forest biomass map

Despite our inability to confirm the spatial patterns of the latest global wall-to-wall forest biomass datasets [5,6] with independent field data [26,27], we concluded that GLAS footprint estimates emerging from the Baccini et al. [5] model offer a large and robust dataset for jurisdictional applications. This GLAS based dataset applied to a set of forest biomass classes also allowed us to track uncertainty at the jurisdictional scale. The large number of GLAS footprint estimates allowed us to identify three landscape variables that correlate with forest biomass (in order of predictive power): 1) disturbance level: primary vs. non-primary forests [29], 2) elevation (100 m threshold), and 3) substrate (basic distinctions between acidic, basic, and anoxic classes) (see S3 File for details). We were surprised to find that a 100 m elevation threshold, not previously described, offers a better predictor of forest biomass than alternative thresholds that have been described at 300 m (lowland to hill dipterocarp transition [33]), ~900 m (used to divide major WWF ecoregions in Borneo), and 1500m (transition to montane cloud forest [35]).

Our emissions estimate would have been 18% higher if we had used a mean value of all GLAS estimates within Berau, rather than identifying optimal biomass strata. This indicates the importance of properly stratified biomass maps. While we conclude that our new biomass benchmark map is the best available for Berau, we note that its spatial predictive power is limited by the resolution of source datasets–particularly the oldest of the three: substrate[30].

### Building a degradation emissions dataset

Accounting for degradation is difficult both because a comprehensive global degradation activity dataset does not yet exist, and because it is often necessary to monitor across three or more impact levels for a given type of degradation (e.g. conventional logging vs. reduced impact vs. none). Given these challenges, we used a hybrid approach that integrates a field inventory dataset [26], government records of logging activity, and the Hansen dataset for haul road impacts. This approach allows for monitoring of emissions reductions achieved by reduced impact logging (RIL-C), which would not be possible using a method that determined felling and skidding impacts as a function of distance from haul roads.

Our results also indicate that substantial source(s) of degradation emissions remain to be well accounted for in Berau. Legal logging explains the loss of 21% of “pre-harvest” (ie. “primary”) forest biomass in Berau [26], or about half of the difference in stocks between “primary” forest and “non-primary” forest classes on oxic soils (Fig C in S3 File). It is not clear whether the full reduction in carbon stocks moving from “primary” and “non-primary” forest classes is due to a sequence of logging harvest events in unsustainable succession (legal or illegal) and/or other potential causes of forest degradation.

### Alternative approaches to jurisdictional accounting

Synthesis of global datasets can be approached with either IPCC stock-difference or gain-loss methods. The stock-difference method is often employed for estimating deforestation emissions [61] because it makes use of existing national forest inventories and national land cover maps. We have encountered a common misunderstanding that the global datasets we used are not consistent with IPCC because they do not generate a land-use change matrix and are not structured for a stock-difference approach. We used the IPCC gain-loss approach here, in keeping with other recent studies of forest loss and degradation [8,26,27,52], because we found this approach better structured both for field-based degradation estimation, and for integrating the latest global datasets of forest loss/gain activity [4,62] and biomass [5,6] datasets.

Our finding that a global forest loss activity dataset [4] and our adaptation of a global forest biomass dataset [5] performed well for the jurisdiction of Berau is a positive signal for effectively nesting jurisdictional accounting within national and international accounting frameworks, and tracking leakage. We recommend that these global datasets are given further consideration by national governments to improve monitoring, reporting, and verification (MRV) systems as part of emerging REDD+ mechanisms. While many countries may prefer to develop proprietary MRV systems, these global datasets offer independent reference data for identifying jurisdictions where improvements to national datasets may be needed, and/or independently verifying the accuracy of national datasets.

Global datasets are not yet available for measuring and monitoring forest degradation fluxes. We recommend field-based methods as referenced here, which could be optionally included as part of national MRV systems for those jurisdictions where degradation fluxes are large.

### Implications of uncertainty analysis

Our estimate of ±22% uncertainty for our net emissions estimate is higher than that reported for prior tier 2–3 estimates. This is an artifact of incomplete accounting of uncertainty for prior estimates. For example, our uncertainty estimate would have been ±1% if we had only accounted for uncertainty in our activity dataset and map-based uncertainty in our biomass dataset (solid inner error bars in Fig 4), as done by others [8]. The most comprehensive prior estimate of overall uncertainty we are aware of [52], stops short of assigning default uncertainty levels to parameters with poorly understood uncertainty–as we have done here. The largest contributions to our overall uncertainty were from parameters which are usually not included in published uncertainty assessments, and are ignored in Tier 1 estimates: decay rate (30% of our total uncertainty range), wetland soil carbon (17%), area of regrowth (11%), and regrowth rate (8%) (Table 3). These often ignored sources of uncertainty are priority research needs in Berau and similar tropical moist forest landscapes in order to better constrain emission estimates. In contrast, the datasets usually commanding the greatest attention—forest loss and forest biomass—contributed small proportions of our overall uncertainty. We attribute the surprisingly small uncertainty associated with our activity dataset and map-based uncertainty in our biomass dataset to the consistently high biomass structure of native dipterocarp forests that dominate Berau’s landscape[26]. This natural context facilitated relatively easy spectral and structural differentiation from anthropogenic non-forest cover types. The context of Berau is in contrast to landscapes with extensive natural transition zones between forest and non-forest ecosystems–which are unavoidable at the very large scales for which higher uncertainty has been reported in recent studies [7,10]. Our map-based uncertainty of carbon density was also mitigated by the number of GLAS footprint estimates (N = 7573). While our analysis uncovers large sources of uncertainty in historic forest carbon emissions that have been generally ignored, this does not necessarily translate to large sources of uncertainty in emissions reductions with respect to setting a historic emissions reference level. As Grassi et al. [3] point out, to the extent that systematic errors are applied to both the historic emissions level and the assessment period level, the error may not affect the difference between the two values. This observation highlights the importance of using the same methods for assessment periods as were used for historic emissions estimates–when used in setting reference emissions levels. Further analysis is needed to understand the extent to which the sources of uncertainty we identified would translate to uncertainty in emissions reductions assessed, and as a function of methods used.

We emphasize the importance of estimating an overall uncertainty range for historic forest carbon flux estimates and including sources of uncertainty that have been previously ignored (e.g. decay rates, regrowth rates, soil carbon emissions). Where necessary, we propose transparently assigning default uncertainty ranges to those variables that do not have a sufficient basis for assigning geography-specific empirical values. This avoids a perverse outcome of ignoring the contribution to overall uncertainty by variables for which uncertainty is less well understood–and often higher. We consider the estimation of overall uncertainty in emissions estimates increasingly important as national and international institutions are challenged with comparing alternative estimates of historic emissions.

## Supporting Information

S1 File Methods and results for accuracy assessment of Hansen forest loss product. (DOCX)

S2 File Complete carbon flux equation. (DOCX)

S3 File Biomass benchmark map methods, results, and comparison with alternatives. (DOCX)

S4 File Legal logging activity methods, and illegal logging emissions methods and results. (DOCX)

S5 File R-code for Monte Carlo simulation. (DOCX)
