# Supplementary material for: Synthesizing Global and Local Datasets to Estimate Jurisdictional Forest Carbon Fluxes in Berau, Indonesia
Source: PLoS One. 2016 Jan 11;11(1):e0146357. doi: 10.1371/journal.pone.0146357 (PMC4709193; doi:10.1371/journal.pone.0146357)
Supplement: S1 File — (DOCX) [file pone.0146357.s001.docx]

# S1 File: Methods and results for accuracy assessment of Hansen forest loss product

We conducted an accuracy assessment of what we judged to be the best available forest loss datasets: (i) the Landsat-based Hansen dataset (1), and (ii) a Landsat-based dataset developed by Forclime for Berau, Malinau, and Kapuas Hulu districts of East Kalimantan (2). Neither of the studies behind these datasets provide accuracy assessment results at the scale of Berau, so we conducted an accuracy assessment on each. To do so, we randomly selected 50 blocks, each 2800-hectares in size (6% of area of Berau, 10% area of SPOT image) (S1 Fig. A).


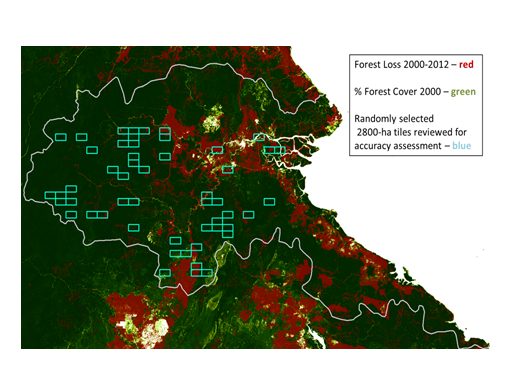


**S1 Fig. A. Location of 50 blocks (2800 ha each) for activity dataset accuracy assessment.** Blocks were randomly selected from available SPOT coverage of Berau – see reference 25 in main text.

In each block we assessed the accuracy of activity data between the years 2000 and 2009 as follows:

1. We confirmed that the area identified as forest loss by activity datasets during the period 2000-2009 had not been previously deforested by visual assessment of Landsat reference imagery from year 2000 (USGS 2009, 30-m resolution).
2. We compared the area identified as forest loss by activity datasets during the period 2000-2009, with SPOT reference imagery for the year 2009 obtained by the Nature Conservancy (10-m resolution, see reference 25 in main text).
3. We mapped and tallied all commission and omission error pixels in each sample block with and generated an error matrix to calculate producers, users, and overall accuracy (3). We then produced standard error and confidence intervals. The Hansen loss data was fed them into the Monte Carlo propagation algorithm for estimating overall uncertainty using the stratified estimator method described by Olofsson *et al.*(4).

The Hansen dataset had a lower, more balanced error range, with 9.2% error of omission and 6.8% error of commission, and virtually all pixels were classified. The Forclime dataset had 22.7% error of omission and 12.8% error of commission (S1 Fig. B). The substantially larger error of omission in the Forclime 2013 dataset appears to be due to a large portion (18%) of the area of Berau that was unclassified in their dataset, and their decision to assume unclassified pixels had no loss. Based on these results, we selected the Hansen dataset for our forest gain and loss estimates, and associated emissions estimates.


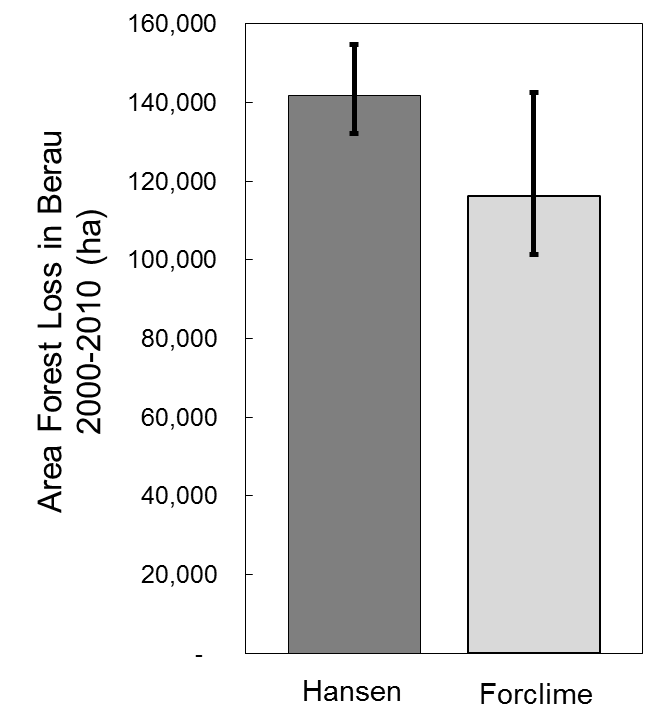


**S1 Fig. B. Results of accuracy assessment** of Hansen *et al.* (1) and Forclime (2) datasets. Error bars represent error of omission (upper bars) and error of commission (lower error bars).

# References

1. Hansen MC, Potapov P V, Moore R, Hancher M, Turubanova S a, Tyukavina a, et al. High-resolution global maps of 21st-century forest cover change. Science [Internet]. 2013;342(6160):850–3. Available from: http://www.ncbi.nlm.nih.gov/pubmed/24233722

2. Navratil P. Land Cover Situation and Land-Use Change in the Districts of West Kalimantan and East Kalimantan, Indonesia. Jakarta, Indonesia; 2013.

3. Congalton RG. A review of assessing the accuracy of classifications of remotely sensed data. Remote Sens Environ. 1991;37(1):35–46.

4. Olofsson P, Foody GM, Stehman S V., Woodcock CE. Making better use of accuracy data in land change studies: Estimating accuracy and area and quantifying uncertainty using stratified estimation. Remote Sens Environ [Internet]. Elsevier Inc.; 2013;129:122–31. Available from: http://dx.doi.org/10.1016/j.rse.2012.10.031
