# Supplementary material for: Synthesizing Global and Local Datasets to Estimate Jurisdictional Forest Carbon Fluxes in Berau, Indonesia
Source: PLoS One. 2016 Jan 11;11(1):e0146357. doi: 10.1371/journal.pone.0146357 (PMC4709193; doi:10.1371/journal.pone.0146357)
Supplement: S2 File — (DOCX) [file pone.0146357.s002.docx]

# S2 File: Complete carbon flux equation

(SA) ${AFd}_{h}= AFd*{FAF}_{h}$

(SB) ${Cd}_{h}= {AGLB}_{h}*CF$

(SC) ${LFd}_{h}={TPFd}_{h}+ {BFd}_{h}*\left( DBF+1-FTF \right)+{DKMd}_{h}*\left\{ {BGBF}_{i}+[1-{FDF}_{h}+CC*{FDF}_{h}]*\left( DBF+1-FTF \right) \right\}$

where

${TPFd}_{h}=$ Post-conversion timber and pulp extraction emissions factor (MgC ha^-1^)

${BFd}_{h}=$ Woody biomass burning emissions factor (MgC ha^-1^)

are calculated as follows:

(SD) ${TPFd}_{h}=FTF*{FPF}_{h}+FTF*\left( 1-{FPF}_{h} \right)*\left( FSWP+FMWP \right)$

(SE) ${BFd}_{h}={FDF}_{h}*\left( 1-CC-CEF \right)$

(SF) $SEFd=FWM*MSLF*SCM+\left( FWHP*SCHP+FWLP*SCLP \right)*\frac{SDDP}{SCDP}$

where

$MSLF=$ Mangrove soil loss factor (% of soil carbon lost) calculated as follows:

(SG) $MSLF=\frac{SDDM}{SCDM}*SCLMT+\left( 1-\frac{SDDM}{SCDM}*SCLMT \right)*SCLMB$

(SH) $EFsl=DBsl*DKMsl+RWsl*\left( FSWP+FMWP \right)*WD*CF$

(SI) $SFr=\left( 1+{BGBF}_{2} \right)*\left[ CSSP*FrH+CSSO*FrO+\left( 1-FrH-FrO \right)*CSSN \right]\div2$

(SJ) $SFl=CSSl*(1+{BGBF}_{2})\div2$. See S2 Table A for description of all parameters.

| **S2 Table A.** Input parameters for emissions equations 1-3 and SA-SJ. For strata-dependent input parameters, maximum and minimum values are shown with mean in parentheses. Assigned uncertainty values are based upon defaults from Table 1. Unassigned uncertainty values were derived from defined empirical distributions from sample data. Percent uncertainty is expressed as the 95% confidence interval divided by the mean. | | | | | | | |  |
| --- | --- | --- | --- | --- | --- | --- | --- | --- |
| **Input Parameter** | **Description** | **Value** | **Units** | **Data source(s)** | **Uncertainty Method** | **% Uncertainty** | **Group** | |
| *Afd* | Area of forest lost in reference period *tp* | 145,488.2 | ha | (1) | Distribution defined from Berau accuracy assessment (this study) | 0.003 | Other | |
| *AGLB_h_* | Aboveground live biomass prior to deforestation stratum *h* | 117 - 404 (281) | Mg ha^-1^ | (2) | Distribution defined from Baccini GLAS model (this study) | 0.008 - 0.109 (0.045) | Forest Carbon | |
| *FAF_h_* | Proportion of forest loss in biomass map stratum *h* | 0.0005 - 0.42 (0.08) | proportion | (3–6) | Distribution defined from GLAS biomass variance (this study) | 0.016 - 0.142 (0.065) | Forest Carbon | |
| *Asl* | Area of active legal commercial logging in HPH during time period *tp* | 93,443 | ha | (7) | Distribution defined from Monte Carlo simulation to fill data gaps | 0.007 | Logging | |
| *Ar* | Area of forest regrowth | 58,524 | ha | (1) | Assigned High | 0.500 | Regrowth | |
| *BGBF_1_* | Belowground live biomass (as % of *Cd*) for all strata with *Cd* > 125 | 0.24 | proportion | (8) | Distribution defined from Mokany global source data | 0.092 | Forest Carbon | |
| *BGBF_2_* | Belowground live biomass (as % of *Cd*) for all strata with *Cd* < 125 | 0.21 | proportion | (8) | Distribution defined from Mokany global source data | 0.344 | Forest Carbon | |
| *CC* | Carbon Retained after combustion of burns within the first dry season. | 0.49 | proportion | (9) | Assigned High | 0.500 | Fire | |
| *SFsl* | Carbon sequestration rate skidding and felling impact areas of logging concessions | 0.37 | Mg C ha^-1^ | (10) | Distribution defined from STREK plot means (n=5) | 1.124 | Regrowth | |
| *CSSN* | Carbon sequestration rate in natural regrowth forests | 3.85 | Mg C ha^-1^ yr^-2^ | (11) | Assigned Medium | 0.200 | Regrowth | |
| **Input Parameter** | **Description** | **Value** | **Units** | **Data source(s)** | **Uncertainty Method** | **95% CI  mean ^-1^** | **Group** | |
| *FrH* | % area regrowth in permitted plantation forests (HTI) | 0.35 | proportion | This study | Assigned High | 0.500 | Regrowth | |
| *CSSP* | Carbon sequestration rate in HTI | 9.80 | Mg C ha^-1^ yr^-2^ | (12,13) | Assigned Low | 0.100 | Regrowth | |
| *FrO* | % area regrowth in oil palm permits | 0.25 | proportion | This study | Assigned Medium | 0.200 | Regrowth | |
| *CSSO* | Carbon sequestration rate of oil palm | 2.97 | Mg C ha^-1^ yr^-2^ | (14) | Assigned High | 0.500 | Regrowth | |
| *DBF* | Dead necromass as a proportion of *Cd* | 0.09 | proportion | (15) | Assigned High | 0.500 | Forest Carbon | |
| *DBsl* | Aboveground dead biomass due to skidding and felling. | 42.8 | Mg C ha^-1^ yr^-1^ | (16) | Distribution defined from East Kalimantan logging concessions (n=9) | 0.164 | Logging | |
| *DKMd_h_* | Post-deforestation model decay Factor for forest stratum *h* | 0.77 - 0.98 (0.83) | proportion | This study, based off of (17) | Assigned High | 0.500 | Decay | |
| *DKMsl* | Post-logging model decay factor | 0.83 | proportion | This study, based off of (17) | Assigned High | 0.500 | Decay | |
| *CEF* | Charcoal (elemental) fraction | 4.0% | proportion | (18) | Assigned High | 0.500 | Fire | |
| *FDF_h_* | Fraction of deforested land burned within following year for forest stratum *h* | 0.004 - 0.73 (0.81) | proportion | This study, employing (7) | Assigned High | 0.500 | Fire | |
| *FWHP* | Fraction of forest land in high peat wetlands | 0.009 | proportion | This study, employing (4) | Assigned Very High | 1.000 | Wetland Soils | |
| *FWLP* | Fraction of deforested area in low peat wetlands | 0.004 | proportion | This study, employing (4) | Assigned Very High | 1.000 | Wetland Soils | |
| *FWM* | Fraction of deforested area in mangrove wetlands | 0.039 | proportion | This study, employing (4) | Assigned Very High | 1.000 | Wetland Soils | |
| *FMWP* | Fraction of roundwood biomass processed into medium-term wood products | 0.082 | proportion | Conversations with HPH managers and (19) | Assigned Medium | 0.200 | Other | |
| **Input Parameter** | **Description** | **Value** | **Units** | **Data source(s)** | **Uncertainty Method** | **95% CI  mean ^-1^** | **Group** | |
| *FPF_h_* | % deforested land where wood products harvested for pulp, for forest stratum *h* | 0.00 - 0.30 (0.14) | proportion | This study, employing (7) | Assigned High | 0.500 | Other | |
| *FSWP* | Fraction of roundwood biomass lost in short-term wood products | 0.24 | proportion | Conversations with HPH managers and (19) | Assigned Medium | 0.200 | Other | |
| *FTF* | Fraction of post-conversion biomass extracted as roundwood | 0.05 | proportion | (20) | Assigned Medium | 0.200 | Other | |
| *RWsl* | Roundwood removed from logging | 37.63 | m^3^ ha^-1^ | Ministry of Forestry reports – see S4 | Distribution defined from Monte Carlo simulation to fill data gaps | 0.230 | Logging | |
| *WD* | Wood density | 0.57 | Mg C m^-3^ | (21) | Distribution defined from Reyes Tropical Asia source data | 0.024 | Other | |
| *SCDM* | Average depth of organic soils in Mangroves | 1.99 | m | (22) | Assigned High | 0.500 | Wetland Soils | |
| *SCDP* | Average depth of organic soils in peat wetlands | 0.75 | m | (4) | Assigned High | 0.500 | Wetland Soils | |
| *SCHP* | Soil carbon in high-peat wetlands prior to deforestation | 302 | Mg C ha^-1^ | (4) | Assigned High | 0.500 | Wetland Soils | |
| *SCLP* | Soil carbon in low-peat wetlands prior to deforestation | 56.66 | Mg C ha^-1^ | (4) | Assigned High | 0.500 | Wetland Soils | |
| *SCM* | Soil carbon in mangroves prior to deforestation | 1,059 | Mg C ha^-1^ | (22) | Distribution defined from Donato Estuarine Mangroves in Borneo (n=5) | 0.156 | Wetland Soils | |
| *SCLMT* | Soil carbon loss in mangroves top 30cm | 0.75 | proportion | (22) | Assigned High | 0.500 | Wetland Soils | |
| *SCLMB* | Soil carbon loss in mangroves below 30cm | 0.35 | proportion | (22) | Assigned High | 0.500 | Wetland Soils | |
| *SDDP* | Average depth of draining occurring on cleared peat soils | 0.60 | m | (23) | Assigned High | 0.500 | Wetland Soils | |
| *SDDM* | Average depth of draining on cleared mangrove soils | 0.30 | m | (22) | Assigned High | 0.500 | Wetland Soils | |
| **Input Parameter** | **Description** | **Value** | **Units** | **Data source(s)** | **Uncertainty Method** | **95% CI  mean ^-1^** | **Group** | |
| *CF* | Carbon fraction | 0.47 | Mg C Mg biomass^-1^ | (24) | Assigned Low | 0.100 | Other | |
| *Tp* | Duration of reference period | 10 | years | This study | None | - | Other | |

# References

1. Hansen MC, Potapov P V, Moore R, Hancher M, Turubanova S a, Tyukavina a, et al. High-resolution global maps of 21st-century forest cover change. Science [Internet]. 2013;342(6160):850–3. Available from: http://www.ncbi.nlm.nih.gov/pubmed/24233722

2. Baccini A, Goetz SJ, Walker WS, Laporte NT, Sun M, Sulla-Menashe D, et al. Estimated carbon dioxide emissions from tropical deforestation improved by carbon-density maps. Nat Clim Chang. 2012;2:182–5.

3. RePPProT. Regional Physical Planning Program for Transmigration Map. Direktorat Bina Program, Indonesia; 1987.

4. Wahyunto S, Subagjo R dan H. Map of Peatland Distribution Area and Carbon Content in Kalimantan 2000-2002. Bogor, Indonesia; 2004.

5. ASTER GDEM v2 [Internet]. Available from: http://gdem.ersdac.jspacesystems.or.jp/

6. Margono B, Potapov P V., Turubanova S, Stolle F, Hansen M. Primary forest cover loss in Indonesia over 2000–2012. Nat Clim Chang. 2014;4:730–5.

7. MoF. Kawasan Hutan (Forest Estate) land use maps [Internet]. General Direktorat of Planning, Indonesia Ministry of Forestry (MoF), prepared by World Resources Institute (2012); 2010. Available from: http://appgis.dephut.go.id/appgis/kml.aspx

8. Mokany K, Raison RJ, Prokushkin AS. Critical analysis of root: Shoot ratios in terrestrial biomes. Glob Chang Biol. 2006;12(1):84–96.

9. Van der Werf GR, Randerson JT, Giglio L, Collatz GJ, Mu M, Kasibhatla PS, et al. Global fire emissions and the contribution of deforestation, savanna, forest, agricultural, and peat fires (1997–2009). Atmos Chem Phys [Internet]. 2010 Dec 10 [cited 2012 Mar 12];10(23):11707–35. Available from: http://www.atmos-chem-phys.net/10/11707/2010/

10. Sist P, Picard N, Gourlet-Fleury S. Sustainable cutting cycle and yields in a lowland mixed dipterocarp forest of Borneo. Ann For Sci. 2003;60(2003):803–14.

11. Bonner MTL, Schmidt S, Shoo LP. A meta-analytical global comparison of aboveground biomass accumulation between tropical secondary forests and monoculture plantations. For Ecol Manage [Internet]. 2013;291:73–86. Available from: http://dx.doi.org/10.1016/j.foreco.2012.11.024

12. Tsai LM. Studies on Acacia mangium in Kemasul Forest, Malaysia. I. Biomass and productivity. J Trop Ecol. 1988;4(03):293.

13. Tsai LM. Biomass and Productivity of 4 - 5 year old Acacia mangium in Sarawak. Peranika. 1986;9:81–7.

14. Thenkabail PS, Stucky N, Griscom BW, Ashton MS, Diels J, van der Meer B, et al. Biomass estimations and carbon stock calculations in the oil palm plantations of African derived savannas using IKONOS data. Int J Remote Sens. 2004;25(23):5447–72.

15. Houghton R a, Lawrence KT, Hackler JL, Brown S. The spatial distribution of forest biomass in the Brazilian Amazon: a comparison of estimates. Glob Chang Biol. 2001;7(7):731–46.

16. Griscom B, Ellis P, Putz FE. Carbon emissions performance of commercial logging in East Kalimantan, Indonesia. Glob Chang Biol. 2014;20(3):923–37.

17. Palace M, Keller M, Hurtt G, Frolking S. A review of above ground necromass in tropical forests. In: Sudarshana P, Nageswara-Rao M, Soneji JR, editors. Tropical Forests. Intech; 2012. p. 215–52.

18. Righi CA, de Alencastro Graça PML, Cerri CC, Feigl BJ, Fearnside PM. Biomass burning in Brazil’s Amazonian “arc of deforestation”: Burning efficiency and charcoal formation in a fire after mechanized clearing at Feliz Natal, Mato Grosso. For Ecol Manage. 2009;258(11):2535–46.

19. Winjum J, Brown S, Schlamadinger B. Forest harvests and wood products: Sources and sinks of atmospheric carbon dioxide. For Sci. 1998;44(2):272–84.

20. Morton DC, Sales MH, Souza CM, Griscom B. Historic emissions from deforestation and forest degradation in Mato Grosso, Brazil: 1) source data uncertainties. Carbon Balance Manag [Internet]. BioMed Central Ltd; 2011 Jan [cited 2012 Jan 2];6(1):18. Available from: http://www.cbmjournal.com/content/6/1/18

21. Reyes G, Brown S, Chapman J, Lugo AE. Wood densities of tropical tree species. New Orleans, Louisiana; 1992.

22. Donato DC, Kauffman JB, Murdiyarso D, Kurnianto S, Stidham M, Kanninen M. Mangroves among the most carbon-rich forests in the tropics. Nat Geosci [Internet]. Nature Publishing Group; 2011;4(5):293–7. Available from: http://dx.doi.org/10.1038/ngeo1123

23. Hooijer a., Page S, Canadell JG, Silvius M, Kwadijk J, Wösten H, et al. Current and future CO_2_ emissions from drained peatlands in Southeast Asia. Biogeosciences [Internet]. 2010 May 12 [cited 2012 Mar 26];7(5):1505–14. Available from: http://www.biogeosciences.net/7/1505/2010/

24. McGroddy M, Daufresne T, Hedin L. Scaling of C: N: P stoichiometry in forests worldwide: implications of terrestrial Redfield-type ratios. Ecology [Internet]. 2004 [cited 2014 Oct 29];85(9):2390–401. Available from: http://www.esajournals.org/doi/abs/10.1890/03-0351
