# Supplementary material for: Synthesizing Global and Local Datasets to Estimate Jurisdictional Forest Carbon Fluxes in Berau, Indonesia
Source: PLoS One. 2016 Jan 11;11(1):e0146357. doi: 10.1371/journal.pone.0146357 (PMC4709193; doi:10.1371/journal.pone.0146357)
Supplement: S3 File — (DOCX) [file pone.0146357.s003.docx]

# S3 File: Biomass benchmark map methods, results, and comparison with alternatives

We derived a regionally refined calibration of the Baccini et al. (1) model for estimating biomass at GLAS lidar footprints based on the relationship between lidar returns and 75 co-located forest inventory plots (40x40 m) collected in SE Asia (S3 Fig. A).


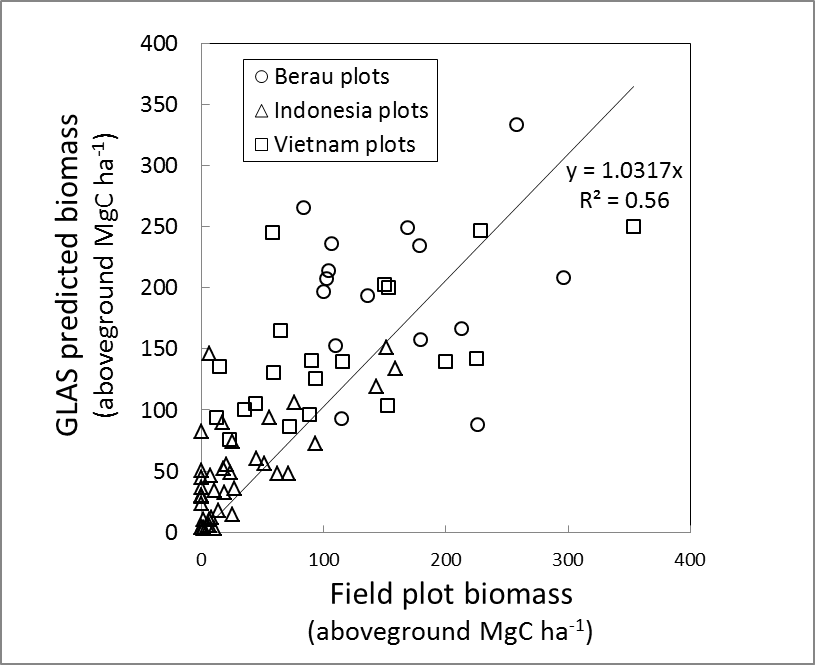


**S3 Fig. A. Relationship between field plot biomass and GLAS estimated biomass.** Forest inventory plots (N=75) were co-located at GLAS lidar footprints in Vietnam (squares), Berau (circles), and other locations within Indonesia (triangles). We observed a highly significant linear relationship (ANOVA) between biomass estimated by filed plots and GLAS lidar footprint returns (R^2^ = 0.37, F (1, 73) = 92.30, P = >0.001).

As part of the process of developing a regionally refined model, we considered alternative allometric equations for relating diameter at breast height measurements of trees in forest inventory plots. We confirmed selection of the Chave et al. (2) allometric equation because it was just below the mean among all equations reviewed (S3 Fig. B), and because the Chave equation using only diameter at breast height (dbh) was only slightly conservative compared the Chave equation that included tree height (see (3) for details).


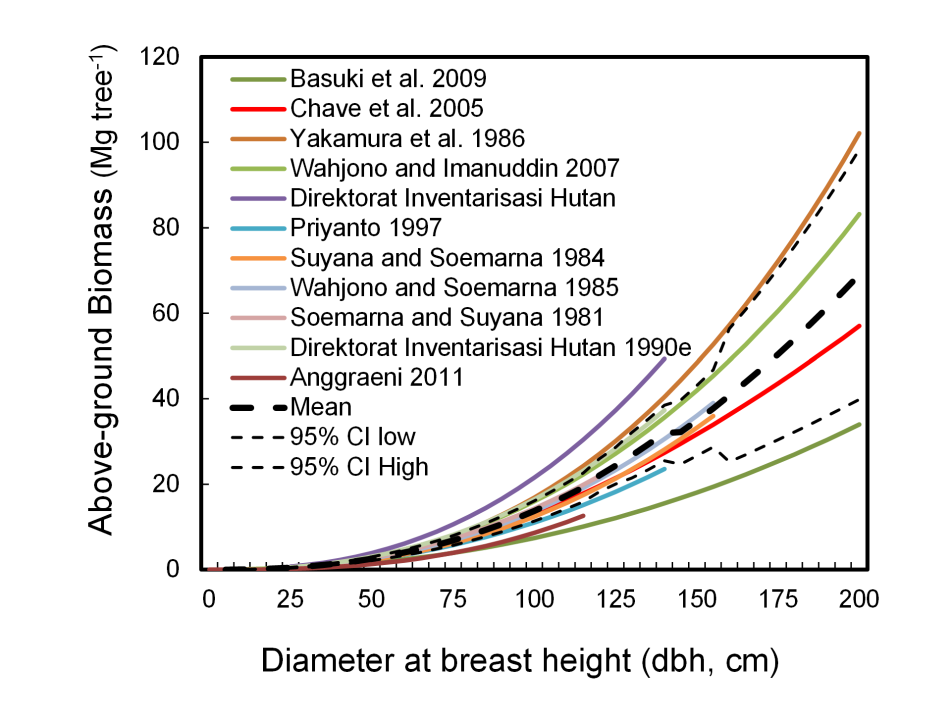


**S3 Fig. B. The implications of selection of alternative allometric equations**. We selected the Chave et al. 2005 allometric equation for deriving above-ground biomass from tree diameters collected from our field inventory data. This equation fell slightly below the mean, and within the 95% confidence intervals, among estimates produced by all equations we considered as relevant to our region.

Based on the regionally refined GLAS lidar model developed, we used GLAS footprint estimates of above-ground forest biomass to test for a difference in mean biomass between “primary” and “non-primary” forest categories (4) using a one-way ANOVA and found a highly significant difference (F(1,7572)=1447.84, P<0.0001). To avoid conflating the influence of anthropogenic and natural variables, we tested for differences in forest biomass among soil categories and among elevation classes on mineral soils using only “primary” forest GLAS values (one-way ANOVA with Tukey’s multiple comparisons). We lumped any soil and elevation strata for which we did not detect significant differences in mean aboveground biomass, with the exception of wetland soil strata classes that warranted different soil carbon emissions assumptions. For the final forest biomass strata (N=13), we calculated average biomass values using GLAS footprint values (Fig. 7, S3 Table A).

After finalizing forest biomass strata, we compared the explanatory power of the most important contrasting levels identified for disturbance (primary vs. non-primary), elevation (0-100m vs. >100m), and soils (acidic vs. non-acidic) for the dominant mineral soil Dipterocarp forests of Berau, using available GLAS footprint estimates (N=6340). The primary vs. non-primary dataset (Margono et al. 2014) explained the largest amount of spatial variability in the dominant mineral soil Dipterocarp forests of Berau. Non-primary forests were 43% lower biomass than primary forests in Dipterocarp forests ((F(1,6338)=2259.23, P<0.0001; S3 Fig. C). Low elevation (0-100m) Dipterocarp forests were 29% lower in biomass than those at higher elevations (F(1,6338)=885.63, P<0.0001; S3 Fig. C). Dipterocarp forests on acidic latisol soils were 14% lower in biomass than those on non-acidic soils (karst, metamorphic, and volcanic), (F(1,6338=67.52, P<0.0001; S3 Fig. C).





**S3 Fig. C. Correlation of biomass with disturbance, elevation and soils.** We compared two levels for three factors: (a) disturbance (primary vs. non-primary), (b) elevation (0-100m vs. >100m), and (c) soils (acidic vs. non-acidic) for the dominant mineral soil Dipterocarp forests of Berau. Biomass values for each factor-level were calculated from GLAS footprint estimates (N=6340). Differences among the two levels were highly significant (P<0.0001, ANOVA) for all three factors (see text for statistical results). Error bars represent 95% confidence intervals.

We lumped all elevation strata above 100 m since we did not detect statistical differences among strata above 100 m. We did observe the same pattern of maximum aboveground forest carbon stocks at 100-900 m elevations for both acidic and non-acidic soil strata (S3 Fig. D).





**S3 Fig. D. Relationships between biomass and strata of elevation and soil.** Although we did not detect a statistical difference in biomass among elevation classes above 100 meters, we did observe a trend of maximum aboveground forest carbon stocks at 100-900 m elevations for both acidic and non-acidic soil strata (a). With regard to soil strata (b), we lumped karst and metamorphic/volcanic strata within mineral (oxic) soils. We also lumped alluvial and coastal peat strata. We maintained separate strata for peat (alluvial and coastal), dense (or deep) peat, and mangroves. Error bars represent 95% confidence intervals.

Likewise, we lumped karst and metamorphic/volcanic soil strata since we did not detect statistical differences among them for primary forests (S3 Fig. D). We maintained distinctions among three wetland soil strata (mangrove, peat, dense peat), despite limited detection of statistical distinction in aboveground biomass, because they invoked different soil carbon emissions assumptions. We lumped two other wetland soil strata (alluvial peat and coastal peat) that did not invoke different soil carbon emissions assumptions.

| **S3 Table A.** Above and belowground carbon stocks, and area change, for 13 forest biomass classes. | | | | | | | |
| --- | --- | --- | --- | --- | --- | --- | --- |
| **Forest Biomass Class Name** | | **Area 2000 (ha)** | **Biomass Carbon Stocks  (AG+BG, MgC)** | **Biomass Carbon Density  (AG+BG, MgC ha^-1^)** | | **Forest loss emissions factor (as % of C density)** | **Area 2010 (ha)** |
| Primary High Fertility Highland | | 59,890 | 15,061,418 | 251 | 82% | | 59,722 |
| Primary High Fertility Lowland | | 158,690 | 36,610,962 | 231 | 90% | | 149,590 |
| Disturbed High Fertility Lowland | | 70,595 | 10,374,611 | 147 | 90% | | 48,576 |
| Primary Low Fertility Highland | | 487,316 | 114,506,284 | 235 | 77% | | 485,609 |
| Primary Low Fertility Lowland | | 1,026,546 | 210,382,292 | 205 | 82% | | 965,979 |
| Disturbed Hi & L Fert. Highland | | 4,579 | 756,638 | 165 | 91% | | 4,508 |
| Disturbed Low Fertility Lowland | | 185,739 | 21,396,079 | 115 | 81% | | 150,957 |
| Primary Mangrove | | 65,070 | 7,773,511 | 119 | 90% | | 60,129 |
| Disturbed Mangrove | | 6,856 | 698,714 | 102 | 91% | | 6,135 |
| Primary Peat | | 16,599 | 2,596,182 | 156 | 92% | | 16,203 |
| Disturbed Peat | | 1,767 | 208,773 | 118 | 93% | | 1,556 |
| Disturbed & Primary Dense Peat | | 6,567 | 676,744 | 103 | 91% | | 5,269 |
| Secondary Forest* | | 12,040 | 860,828 | 71 | 88% | | 2,532 |
| **Total:** | **2,102,255** | | **427,716,960** | **196** | **84%** | | **2,038,085** |

*area of secondary forest in year 2010 does not include secondary forest which emerged after 2000.

# References

1. Baccini A, Goetz SJ, Walker WS, Laporte NT, Sun M, Sulla-Menashe D, et al. Estimated carbon dioxide emissions from tropical deforestation improved by carbon-density maps. Nat Clim Chang [Internet]. 2012;2:182–5. Available from: http://eorder.sheridan.com/3_0/display/index.php?flashprint=1608

2. Chave J, Andalo C, Brown S. Tree allometry and improved estimation of carbon stocks and balance in tropical forests. Oecologia [Internet]. 2005 [cited 2012 Aug 5];145:87–99. Available from: http://www.springerlink.com/index/p1k67p2175l56365.pdf

3. Griscom B, Ellis P, Putz FE. Carbon emissions performance of commercial logging in East Kalimantan, Indonesia. Glob Chang Biol. 2014;20(3):923–37.

4. Margono B, Potapov P V., Turubanova S, Stolle F, Hansen M. Primary forest cover loss in Indonesia over 2000–2012. Nat Clim Chang. 2014;4:730–5.
