# Supplementary material for: Synthesizing Global and Local Datasets to Estimate Jurisdictional Forest Carbon Fluxes in Berau, Indonesia
Source: PLoS One. 2016 Jan 11;11(1):e0146357. doi: 10.1371/journal.pone.0146357 (PMC4709193; doi:10.1371/journal.pone.0146357)
Supplement: S4 File — (DOCX) [file pone.0146357.s004.docx]

# S4 File: Legal logging activity methods, and illegal logging emissions methods and results

We attempted to develop a remote-sensing dataset of logging activity for Berau (CLASlite, (1)) and concluded that it was not feasible due to persistent cloud cover in Berau as represented in available Landsat imagery and considering that the limited time window for detecting the signal of selective logging. We thus concluded that there are currently no passive remote sensing methods for measuring or monitoring skidding and felling activity at the scale of Berau with sufficient accuracy. Some studies have used haul road construction as an overall indicator of logging activity (2–4). We concluded that this approach was insufficient for our carbon accounting purposes because (i) it does not capture felling and skidding operations that use preexisting haul roads (frequently observed in Berau region by Griscom *et al.* (5)), and (ii) it is not able to differentiate haul roads within an annual cutting block (proximate to felling and skidding) from haul roads not proximate to felling and skidding activity (e.g. extensive logging access roads, or roads for purposes other than logging such as coal mining and initiation of oil palm conversion). Thus we made a conservative assumption that commercial logging skidding and felling activity is limited to logging reported by the government, which we refer to as “legal logging.” For the 43% of the concession-years for which we were unable to acquire logging records, we estimated harvest levels based on total concession area and the average harvest rates in concession-years with records (S4 Table A).

We suspect that illegal logging may be a substantial source of unquantified emissions. We generated a preliminary estimate of illegal logging emissions by applying our field-based estimate of emissions per unit timber volume (5) to the mean of reported values for the volume of timber generated from Berau by illegal logging (6,7). With this approach, we estimated that about 336,000 m^3^ of wood gerenating 1 Tg CO^2^ are produced by illegal logging each year, which would represent a 9% increase in gross forest carbon emissions in Berau. We do not include this illegal logging emissions estimate in our overall emissions estimates because we do not understand the empirical basis for the methods described by Obidzinsky and Barr (6) and Obidzinski and Andrianto (7). We also do not know whether it is appropriate to apply our estimate of emissions per unit timber volume to their illegal harvest volume figures.

| **S4 Table A**. Government of Indonesia records for area harvested (ha) by year in 20 active logging concessions (IUPHHK-HA) in Berau. Numbers in *italics* were concession-years with missing government records, so values were estimated based on total concession area and harvest rates of concession-years with data. Names of concession owners have been replaced with letter codes (A, B, C…). | | | | | | | | | | | | | | |
| --- | --- | --- | --- | --- | --- | --- | --- | --- | --- | --- | --- | --- | --- | --- |
| **Concession** | **Area (ha)** | **2000** | **2001** | **2002** | **2003** | **2004** | **2005** | **2006** | **2007** | **2008** | **2009** | **2010** | **2011** | **2012** |
| A | 137,790 | *2046* | *2046* | *2046* | 0 | 0 | 0 | 0 | 1102 | 1790 | 1056 | 1526 | 1308 | 1250 |
| B | 98,611 | *1465* | *1465* | *1465* | 0 | 0 | 0 | *809* | *1433* | *1603* | *1127* | *1765* | *2223* | *1465* |
| C | 42,625 | 611 | 219 | 1090 | 971 | 657 | 834 | 537 | 527 | 183 | 0 | 0 | 682 | *633* |
| D | 47,763 | 0 | 780 | 1114 | 222 | 431 | 64 | 164 | 258 | 182 | 688 | 861 | 1136 | *709* |
| E | 48,438 | *549* | *549* | *549* | *549* | *549* | *549* | 560 | 583 | 822 | 194 | 194 | 292 | *719* |
| F | 46,080 | 1200 | 1200 | 1900 | 1200 | 1200 | 1180 | 794 | 872 | 672 | 620 | 909 | 907 | *684* |
| G | 25,630 | *291* | *291* | *291* | *291* | *291* | *291* | *291* | *291* | *291* | 503 | 592 | 697 | *381* |
| H | 27,684 | *314* | *314* | *314* | *314* | *314* | *314* | *314* | *314* | *314* | *411* | 540 | 719 | *411* |
| I | 58,482 | 258 | 421 | 716 | 108 | 27 | 160 | 740 | 804 | 557 | 354 | 0 | 0 | *869* |
| J | 103,178 | 126 | 0 | 0 | 1532 | 958 | 1532 | 1047 | 1744 | 854 | 456 | 791 | 0 | 0 |
| K | 49,070 | 0 | 0 | 167 | 0 | 0 | 169 | 346 | 516 | 310 | 236 | 432 | 0 | 0 |
| L | 37,667 | 483 | 883 | 285 | 303 | 434 | 318 | 567 | 318 | 99 | 195 | 195 | 0 | 0 |
| M | 65,934 | *979* | *979* | *979* | 0 | 0 | 0 | 0 | *979* | *979* | 1572 | 489 | 1135 | 331 |
| N | 35,927 | *534* | *534* | *534* | 0 | 0 | 0 | 0 | *534* | *534* | *534* | *534* | *534* | *534* |
| O | 8,201 | *93* | *93* | *93* | *93* | *93* | *93* | *93* | *93* | *93* | 299 | 0 | 478 | *122* |
| P | 46,162 | *523* | *523* | *523* | *523* | *523* | *523* | *523* | *523* | *523* | 410 | 113 | 0 | 0 |
| Q | 10,844 | *123* | *123* | *123* | *123* | *123* | *123* | *123* | *123* | *123* | 14 | 54 | 63 | 133 |
| R | 19,325 | *219* | *219* | *219* | *219* | *219* | *219* | *219* | *219* | *219* | 642 | 405 | 515 | 48 |
| S | 13,367 | *152* | *152* | *152* | *152* | *152* | *152* | *152* | *152* | *152* | 53 | 22 | 220 | 479 |
| T | 8,760 | *99* | *99* | *99* | *99* | *99* | *99* | *99* | *99* | *99* | 204 | 201 | 249 | 97 |
| ***Total area harvested (ha)*** | ***931,538*** | ***10,065*** | ***10,889*** | ***12,659*** | ***6,699*** | ***6,070*** | ***6,619*** | ***7,378*** | ***11,484*** | ***10,399*** | ***9,569*** | ***9,622*** | ***11,158*** | ***8,864*** |

# References

1. Asner GP, Knapp DE, Broadbent EN, Oliveira PJC, Keller M, Silva JN. Selective logging in the Brazilian Amazon. Science (80- ). 2005;310(5747):480–2.

2. Margono B, Potapov P V., Turubanova S, Stolle F, Hansen M. Primary forest cover loss in Indonesia over 2000–2012. Nat Clim Chang. 2014;4:730–5.

3. Bryan JE, Shearman PL, Asner GP, Knapp DE, Aoro G, Lokes B. Extreme Differences in Forest Degradation in Borneo: Comparing Practices in Sarawak, Sabah, and Brunei. PLoS One. 2013;8(7).

4. Laporte NT, Stabach J a, Grosch R, Lin TS, Goetz SJ. Expansion of industrial logging in Central Africa. Science. 2007;316(5830):1451.

5. Griscom B, Ellis P, Putz FE. Carbon emissions performance of commercial logging in East Kalimantan, Indonesia. Glob Chang Biol. 2014;20(3):923–37.

6. Obidzinski K, Barr C. The effects decentralisation on forests and forest industries in Berau district, East Kalimantan. Decentralisation of forest administration in Indonesia: Implications for forest sustainability, community livelihoods, and economic development [Internet]. Jakarta, Indonesia; 2003. Available from: http://www.cifor.org/publications/pdf_files/Books/Decentralisation-Case9.pdf

7. Obidzinski K, Andrianto A. Illegal Forestry Activities in Berau and East Kutai Districts, East Kalimantan: Impacts on Economy, Environment, and Society. Forest Governance Program, Center for International Forestry Research (CIFOR). Bogor Barat , Indonesia; 2005.
