# Supplementary material for: Synthesizing Global and Local Datasets to Estimate Jurisdictional Forest Carbon Fluxes in Berau, Indonesia
Source: PLoS One. 2016 Jan 11;11(1):e0146357. doi: 10.1371/journal.pone.0146357 (PMC4709193; doi:10.1371/journal.pone.0146357)
Supplement: S5 File — (DOCX) [file pone.0146357.s005.docx]

# S5 File: R-code for Monte Carlo simulation

#######################################################################

# Monte Carlo simulation of equation for estimating net LULUCF Carbon emissions

# in Berau, Indonesia 2000-2010

#

# Author(s):

# Peter Ellis

# The Nature Conservancy

# Arlington, VA 20712 USA

# email: pellis@tnc.org

#

# Jeffrey S. Evans

# The Nature Conservancy and

# University of Wyoming

# Laramie, WY 82070 USA

# email: jeffrey_evans@tnc.org

#

# Citation:

# Griscom, B.W., P.W. Ellis, A. Baccini, D. Marthinus, J.S. Evans, Ruslandi (In Review) # Synthesizing global and local datasets to estimate jurisdictional forest carbon fluxes in # Berau, Indonesia. PLoS-One

#######################################################################

#

# Block 1 - mean and standard deviation values used in simulations

# stored in "ic" and "cover.coef" data.frame objects

#

#######################################################################

ic <- data.frame(row.names=c("AD_t","AL_t","AR_t","CC","CSSl","CSSn","FRh","CSSp",

"FRo","CSSo","DBF","DBL","DKmL","EF","FFHP","FFLP","FFM","FMWP","FSWP","FTF","RWl",

"WD","SCDM","SCDP","SCHP","SCLP","SCM","SCLmT","SCLmB","SDDp","SDDm","CF","tp",

"CO_two","PLS","PGLR"),

mean=c(145488.20520, 93442.60219, 58523.68765, 0.49000, 0.36836, 3.85000,

0.35044, 9.80000, 0.24845, 2.97000, 0.09000, 42.82056, 0.82712, 0.04000,

0.00892, 0.00417, 0.03891, 0.08244, 0.24000, 0.05000, 37.62853,0.57000,

1.99440, 0.75000, 301.91000, 56.66000, 1059.18000, 0.75000, 0.35000, 0.60000,

0.30000, 0.47000, 10.00000, 3.66667, 0.29206, 0.044278249),

sd=c(196.17417, 325.36164, 14929.51216, 0.12500, 0.21116, 0.39286, 0.08940,

0.50000, 0.02535, 0.75765, 0.02296, 3.58336, 0.21100, 0.01020, 0.00455, 0.00213,

0.01985, 0.00841, 0.02449, 0.00510, 4.41020, 0.00700, 0.50878, 0.19133, 77.01786,

14.45408, 84.47636, 0.19133, 0.08929, 0.15306, 0.07653, 0.02398, 0.00010, 0.00010,

0.00010, 0.00010),

description=c(

"Area of forest lost in reference period tp",

"Area of active legal commercial logging in HPH during timp period tp",

"Area of forest regrowth",

"Carbon Retained after combustion of burns within the first dry season",

"Carbon sequestration rate skidding and felling impact areas of logging concessions",

"Carbon sequestration rate in \"natural\" secondary forests",

"% area regrowth in HTI Permit",

"Carbon sequestration rate in permitted plantation forests (HTI)",

"% area regrowth in oil palm permits",

"Carbon sequestration rate of oil palm",

"Amount of AGDB as a proportion of AGLB",

"Aboveground dead biomass due to skidding and felling",

"Post-dogging model decay factor",

"Charcoal (elemental) fraction",

"Fraction of forest land in high peat wetlands",

"Fraction of Deforested Area in low peat wetlands",

"Fraction of deforested area in mangrove wetlands",

"Fraction of roundwood biomass processed into medium-term wood products",

"Fraction of RWB lost in short-term wood products",

"Fraction of roundwood biomass from Defor. timber extraction",

"Roundwood removed from logging",

"Wood density",

"Average depth of organic soils in Mangroves",

"Average depth of organic soils in peat wetlands",

"Soil carbon in high-peat wetlands prior to deforestation",

"Soil carbon in low-peat wetlands prior to deforestation",

"Soil carbon in mangroves prior to deforestation",

"Soil carbon loss in mangroves top 30cm",

"Soil carbon loss in mangroves below 30cm",

"Average depth of draining occurring on cleared peat soils",

"Average depth of draining on cleared mangrove soils",

"Carbon fraction",

"Duration of reference period",

"Carbon Dioxide Equivalent conversion factor",

"Proportion of Skidding/Felling that is skidding",

"% of gain pixels in logging roads") )

cover.coef <- data.frame(row.names = c(

"Primary High Fertility Highland","Primary High Fertility Lowland",

"Disturbed High Fertility Lowland","Primary Low Fertility Highland",

"Primary Low Fertility Lowland ","Disturbed High and Low Fertility Highland",

"Disturbed Low Fertility Lowland","Primary Mangrove",

"Disturbed Mangrove","Primary Peat",

"Disturbed Peat","Disturbed & Primary Dense Peat",

"Secondary Forest"),

AGLB.mean = c(403.83204, 370.46522, 235.98384, 377.31579, 329.09188,

265.33183, 184.97694, 191.83194, 163.65974, 251.14697, 189.70980,

165.47516, 117.46637),

AGLB.sd = c(9.90879, 3.58371, 3.62291, 3.40549, 1.40926, 11.06212,

1.33652, 2.70680, 5.07649, 9.45958, 10.62030, 5.34459, 1.85400),

Amap.mean = c(0.00116, 0.06255, 0.15135, 0.01173, 0.41630, 0.00049,

0.23907, 0.03396, 0.00495, 0.00272, 0.00145, 0.00892, 0.06536),

Amap.sd = c(0.00002, 0.00163, 0.00537, 0.00010, 0.00460, 0.00001,

0.00304, 0.00074, 0.00021, 0.00015, 0.00010, 0.00040, 0.00475),

DKmD.mean = c(0.82501, 0.91535, 0.91663, 0.77241, 0.79610, 0.93430,

0.77842, 0.91996, 0.93943, 0.95003, 0.98142, 0.93690, 0.88671),

DKmD.sd = c(0.21046, 0.23351, 0.23383, 0.19704, 0.20309, 0.23834,

0.19858, 0.23468, 0.23965, 0.24235, 0.25036, 0.23901, 0.22620),

FDF.mean = c(0.33063, 0.71224, 0.69508, 0.27474, 0.86855, 0.58493,

0.82413, 0.99582, 0.92081, 0.84080, 0.97873, 0.82330, 0.78807),

FDF.sd = c(0.08434, 0.18169, 0.17732, 0.07009, 0.22157, 0.14922,

0.21024, 0.25404, 0.23490, 0.21449, 0.24968, 0.21002, 0.20104),

FPF.mean=c(0.17981, 0.25966, 0.30422, 0.00799, 0.05521, 0.02192, 0.16299,

0.00318, 0.07757, 0.19419, 0.16041, 0.16041, 0.19656),

FPF.sd=c(0.04587, 0.06624, 0.07761, 0.00204, 0.01408, 0.00559, 0.04158,

0.00081, 0.01979, 0.08417, 0.04954, 0.04092, 0.05014),

BGBF.mean=c(0.23500, 0.23500, 0.23500, 0.23500, 0.23500, 0.23500, 0.23500,

0.23500, 0.23500, 0.23500, 0.23500, 0.23500, 0.20500),

BGBF.sd=c(0.01100, 0.01100, 0.01100, 0.01100, 0.01100, 0.01100, 0.01100,

0.01100, 0.01100, 0.01100, 0.01100, 0.01100, 0.03600))

print(ic[,1:2])

print(cover.coef)

#######################################################################

#

# Block 2 - Monte Carlo Simulation Function

#

#######################################################################

bfcp.monte.carlo <- function(n, echo = TRUE) {

library(Runuran)

i = 0

# list object used to store simulations

results <- list(delta_SCd = vector(), delta_WCd = vector(),

delta_Cd_t = vector(),delta_Cl_t = vector(),

delta_Cs_t = vector(), delta_Cg_t = vector(),

delta_C_t = vector())

for(i in 1:n) {

if(echo) cat("iteration", i, "\n")

## Equation constants, with error, drawn from normal distributions

AD_t <- urnorm(1, mean = ic["AD_t", "mean"], sd = ic["AD_t", "sd"], lb = 0)

AL_t <- urnorm(1, mean = ic["AL_t", "mean"], sd = ic["AL_t", "sd"], lb = 0)

AR_t <- urnorm(1, mean = ic["AR_t", "mean"], sd = ic["AR_t", "sd"], lb = 0)

CC <- urnorm(1, mean = ic["CC", "mean"], sd = ic["CC", "sd"], lb = 0)

CSSl <- urnorm(1, mean = ic["CSSl", "mean"], sd = ic["CSSl", "sd"], lb = 0)

CSSn <- urnorm(1, mean = ic["CSSn", "mean"], sd = ic["CSSn", "sd"], lb = 0)

FRh <- urnorm(1, mean = ic["FRh", "mean"], sd = ic["FRh", "sd"], lb = 0)

CSSp <- urnorm(1, mean = ic["CSSp", "mean"], sd = ic["CSSp", "sd"], lb = 0)

FRo <- urnorm(1, mean = ic["FRo", "mean"], sd = ic["FRo", "sd"], lb = 0)

CSSo <- urnorm(1, mean = ic["CSSo", "mean"], sd = ic["CSSo", "sd"], lb = 0)

BGBF = urnorm(1, mean = cover.coef[,"BGBF.mean"][1], sd = cover.coef[,"BGBF.sd"][1], lb = 0)

BGBFs = urnorm(1, mean = cover.coef[,"BGBF.mean"][13], sd = cover.coef[,"BGBF.sd"][13], lb = 0)

DBF <- urnorm(1, mean = ic["DBF", "mean"], sd = ic["DBF", "sd"], lb = 0)

DBL <- urnorm(1, mean = ic["DBL", "mean"], sd = ic["DBL", "sd"], lb = 0)

DKmL <- urnorm(1, mean = ic["DKmL", "mean"], sd = ic["DKmL", "sd"], lb = 0)

EF <- urnorm(1, mean = ic["EF", "mean"], sd = ic["EF", "sd"], lb = 0)

FFHP <- urnorm(1, mean = ic["FFHP", "mean"], sd = ic["FFHP", "sd"], lb = 0)

FFLP <- urnorm(1, mean = ic["FFLP", "mean"], sd = ic["FFLP", "sd"], lb = 0)

FFM <- urnorm(1, mean = ic["FFM", "mean"], sd = ic["FFM", "sd"], lb = 0)

FMWP <- urnorm(1, mean = ic["FMWP", "mean"], sd = ic["FMWP", "sd"], lb = 0)

FSWP <- urnorm(1, mean = ic["FSWP", "mean"], sd = ic["FSWP", "sd"], lb = 0)

FTF <- urnorm(1, mean = ic["FTF", "mean"], sd = ic["FTF", "sd"], lb = 0)

RWl <- urnorm(1, mean = ic["RWl", "mean"], sd = ic["RWl", "sd"], lb = 0)

WD <- urnorm(1, mean = ic["WD", "mean"], sd = ic["WD", "sd"], lb = 0)

SCDM <- urnorm(1, mean = ic["SCDM", "mean"], sd = ic["SCDM", "sd"], lb = 0)

SCDP <- urnorm(1, mean = ic["SCDP", "mean"], sd = ic["SCDP", "sd"], lb = 0)

SCHP <- urnorm(1, mean = ic["SCHP", "mean"], sd = ic["SCHP", "sd"], lb = 0)

SCLP <- urnorm(1, mean = ic["SCLP", "mean"], sd = ic["SCLP", "sd"], lb = 0)

SCM <- urnorm(1, mean = ic["SCM", "mean"], sd = ic["SCM", "sd"], lb = 0)

SCLmT <- urnorm(1, mean = ic["SCLmT", "mean"], sd = ic["SCLmT", "sd"], lb = 0)

SCLmB <- urnorm(1, mean = ic["SCLmB", "mean"], sd = ic["SCLmB", "sd"], lb = 0)

SDDp <- urnorm(1, mean = ic["SDDp", "mean"], sd = ic["SDDp", "sd"], lb = 0)

SDDm <- urnorm(1, mean = ic["SDDm", "mean"], sd = ic["SDDm", "sd"], lb = 0)

CF <- urnorm(1, mean = ic["CF", "mean"], sd = ic["CF", "sd"], lb = 0)

CO_two = urnorm(1, mean = ic["CO_two", "mean"], sd = ic["CO_two", "sd"], lb = 0)

tp = 10 + runif(1, -sd(c(10-1,10+1))/2, sd(c(10-1,10+1))/2)

#########################

# Vector-based terms of equation

# Terms, stratified by the 13 covertypes (defined in cover.coef),

# drawn from normal distribution

for(i in c("AGLB", "Amap", "DKmD", "FDF", "FPF", "BGBF")) {

for(j in 1:13) {

assign(paste0(i,j), urnorm(1, mean = cover.coef[,paste0(i,".mean")][j],

sd = cover.coef[,paste0(i,".sd")][j]))

}

}

for(j in 1:13) { assign(paste0("TPFd",j), (FTF * get(paste0("FPF",j))) + ((FTF *

(1-get(paste0("FPF",j)))) * (FSWP + FMWP))) }

for(j in 1:13) { assign(paste0("BFd",j), get(paste0("FDF",j)) * (1 - CC - EF)) }

for(j in 1:13) { assign(paste0("DLF",j), get(paste0("TPFd",j)) + ( get(paste0("BFd",j)) *

(DBF + (1 - FTF))) + (get(paste0("DKmD",j)) *

(BGBF + (((1 - get(paste0("FDF",j))) + (CC * get(paste0("FDF",j)))) *

((DBF + (1 - FTF))))))) }

DLF13 <- (TPFd13)+(BFd13*(DBF+(1-FTF)))+(DKmD13*(BGBFs+(((1-FDF13)+(CC*FDF13))*((DBF+(1-FTF))))))

#########################

# Carbon equation

delta_WCd <- CF * AD_t * ( Amap1 * AGLB1 * DLF1 + Amap2 * AGLB2 * DLF2 + Amap3 *

AGLB3 * DLF3 + Amap4 * AGLB4 * DLF4 + Amap5 * AGLB5 * DLF5 + Amap6 *

AGLB6 * DLF6 + Amap7 * AGLB7 * DLF7 + Amap8 * AGLB8 * DLF8 + Amap9 *

AGLB9 * DLF9 + Amap10 * AGLB10 * DLF10 + Amap11 * AGLB11 * DLF11 +

Amap12 * AGLB12 * DLF12 + Amap13 * AGLB13 * DLF13 )

MSLF <- ((SDDm / SCDM) * SCLmT) + ((1 - ((SDDm / SCDM) * SCLmT)) * SCLmB)

delta_SCd <- AD_t * ((FFM * MSLF * SCM) + (((FFHP * SCHP) + (FFLP * SCLP)) * (SDDp / SCDP)))

delta_Cd_t <- delta_WCd + delta_SCd

SFL <- DBL * DKmL

WPL <- RWl * (FSWP + FMWP) * WD * CF

delta_Cl_t <- AL_t * (SFL + WPL)

CSd <- AR_t * tp / 2 * (1 + BGBF) * ((CSSp * FRh) + (CSSo * FRo) + ((1 - FRh - FRo) * CSSn))

CSl <- AL_t * CSSl * (1 + BGBFs) * (tp / 2)

delta_Cs_t <- CSd + CSl

# Accumlate simulations for delta_C_t (Net C-Flux (Tg CO2/yr)) and other estimates

results[["delta_SCd"]] <- append(results[["delta_SCd"]], delta_SCd)

results[["delta_WCd"]] <- append(results[["delta_WCd"]], delta_WCd)

results[["delta_Cd_t"]] <- append(results[["delta_Cd_t"]], delta_Cd_t)

results[["delta_Cl_t"]] <- append(results[["delta_Cl_t"]], delta_Cl_t)

results[["delta_Cs_t"]] <- append(results[["delta_Cs_t"]], delta_Cs_t)

results[["delta_Cg_t"]] <- append(results[["delta_Cg_t"]], (delta_Cd_t + delta_Cl_t) )

results[["delta_C_t"]] <- append(results[["delta_C_t"]], ((delta_Cd_t + delta_Cl_t -

delta_Cs_t) ) )

}

return(results)

}

#######################################################################

#

# Block 3 - Run Monte Carlo Simulation and plot results.

# Simulation results stored in "mc.sim" list object

# Summary results stored in "uncertanty" data.frame object

#

#######################################################################

# Standard error function

se <- function(x) sqrt(var(x)/length(x))

# Run Monte Carlo simulation with n=100,000 replicates

mc.sim <- bfcp.monte.carlo(100000)

#mc.sim <- bfcp.monte.carlo(100) # test

v = c("delta_C_t", "delta_Cd_t", "delta_Cl_t", "delta_Cs_t", "delta_Cg_t", "delta_SCd")

uncertanty <- data.frame()

for(i in v){

uncertanty <- rbind(uncertanty, c(min(mc.sim[[i]]), mean(mc.sim[[i]]),

max(mc.sim[[i]]), sd(mc.sim[[i]]), se(mc.sim[[i]])) )

}

names(uncertanty) <- c("min", "mean", "max", "sd", "std.err")

row.names(uncertanty) <- v

# Display numeric results

print(uncertanty)

# Write simulation results to csv file

write.csv(uncertanty, "BFCP_mc.csv", row.names = TRUE)

# Plot simulation distribution with mean estimate

mc.den <- density(mc.sim[["delta_C_t"]])

plot(mc.den, main="Estimated Net LULUCF C Emissions - Berau,

Indonesia 2000-2010", type="n", xlab="TgCo2 yr-1",

ylab="Probability Density Function")

polygon(density(mc.sim[["delta_C_t"]]), col="blue")

abline(v = mean(mc.sim[["delta_C_t"]]))

# text(80, 0.03, bquote(bar(x) == .(round(mean(mc.sim[["delta_C_t"]]),3))))

# text(80, 0.028, bquote(sigma == .(round(sd(mc.sim[["delta_C_t"]]),3))))

# text(80, 0.026, paste("SE = ", round(se(mc.sim[["delta_C_t"]]),3), sep=""))

save.image("BFCP_MCsimulation.MC.RData")

############################### END CODE #############################
